# Combined inhibition of HER2 and VEGFR synergistically improves therapeutic efficacy via PI3K-AKT pathway in advanced ovarian cancer

**DOI:** 10.1186/s13046-024-02981-5

**Published:** 2024-02-26

**Authors:** Weisong Li, Kai Zhang, Wenjun Wang, Yuanyuan Liu, Jianming Huang, Meihong Zheng, Ling Li, Xinyu Zhang, Minjuan Xu, Guofang Chen, Liefeng Wang, Shuyong Zhang

**Affiliations:** 1 Department of Pathology, First Affiliated Hospital, Gannan Medical University, Ganzhou, 341000 China; 2 Key Laboratory of Prevention and Treatment of Cardiovascular and Cerebrovascular Diseases (Ministry of Education), Gannan Medical University, 1 Hexie Road, Rongjiang New District, Ganzhou, 341000 China; 3 School of Basic Medicine, Gannan Medical University, Ganzhou, 341000 China; 4 Department of Immunology, School of Basic Medical Sciences, Fudan University, Shanghai, 200032 China; 5 Department of Gynaecology and Obstetrics, Ganzhou People’s Hospital (The Affiliated Ganzhou Hospital of Nanchang University), Ganzhou, 341000 China; 6 Shanghai Key Laboratory of Maternal Fetal Medicine, Clinical and Translational Research Center, Shanghai First Maternity and Infant Hospital, School of Medicine, Shanghai Institute of Maternal-Fetal Medicine and Gynecologic Oncology, Tongji University, Shanghai, 200092 China

**Keywords:** HER2, VEGFR, Antibody drug conjugate, RC48, Cediranib Maleate, Synergetic effect, Ovarian cancer

## Abstract

**Background:**

Ovarian cancer (OC) is a prevalent malignancy in the female reproductive system, and developing effective targeted therapies for this disease remains challenging. The aim of this study was to use clinically-relevant OC models to evaluate the therapeutic effectiveness of RC48, an antibody-drug conjugate (ADC) targeting HER2, either alone or in combination with the VEGFR inhibitor Cediranib Maleate (CM), for the treatment of advanced OC.

**Methods:**

OC tumor specimens and cell lines were analyzed to determine HER2 and VEGFR expression by Western blot, immunocytochemistry and immunofluorescence. Moreover, the OC cell lines, cell-derived xenograft (CDX) and patient-derived xenograft (PDX) models were treated with RC48 and/or CM and then subjected to cell proliferation, viability, apoptosis, and tumor growth analyses to evaluate the feasibility of combination therapy for OC both in vitro and in vivo. Additionally, RNA-Seq was performed to investigate the critical mechanism underlying the combination therapy of RC48 and CM.

**Results:**

Our results demonstrated that RC48 alone effectively targeted and inhibited the growth of HER2-positive OC tumors in both cell lines and PDX models. Furthermore, the combination of RC48 and CM synergistically induced tumor regression in human OC cell lines, as well as CDX and PDX models. Mechanistically, we observed that the combination treatment inhibited the growth of OC cells involved inducing apoptosis and suppressing cell motility. RNA-seq analysis provided further mechanistic insights and revealed that co-administration of RC48 and CM downregulated multiple cancer-related pathways, including the AKT/mTOR pathway, cell cycle, and cell proliferation. Notably, our data further confirmed that the PI3K-AKT pathway played a key role in the inhibition of proliferation triggered by combinational treatment of RC48 and CM in OC cells.

**Conclusions:**

These findings provide a preclinical framework supporting the potential of dual targeting HER2 and VEGFR as a promising therapeutic strategy to improve outcomes in patients with OC.

**Supplementary Information:**

The online version contains supplementary material available at 10.1186/s13046-024-02981-5.

## Introduction

Ovarian cancer (OC) is a prevalent malignancy in the female reproductive system, and it has the highest mortality rate among gynecological cancers [1–3]. The overall 5-year survival rate for OC is about 48.6%. However, it is noteworthy that only 15.7% of OC cases are found at the local stage, where the 5-year survival rate reaches 92.6%. In contrast, 58% of OC cases are diagnosed at the metastasized stage, leading to a sharp decline in the 5-year survival rate to 30.2% [4, 5]. Currently, there are few drugs available for targeted therapy in OC, with only two drugs-vascular endothelial growth factor receptors (VEGFRs) and poly (ADP-ribose) polymerase (PARP) inhibitors used in first-line maintenance therapy [6, 7]. There is still no effective treatment for recurrent and platinum-resistant OC. For example, the response rate of recurrent patients receiving chemotherapy is less than 20%, the median progression-free survival (PFS) is less than 4 months, and the median overall survival (OS) is less than 12 months. The duration of response to non-platinum chemotherapy in patients with platinum-resistant OC is only 3–7 months [8–10]. In addition, single-targeted treatments often fail to achieve the desired therapeutic outcomes [11]. Thus, finding new and effective therapeutic strategies, especially the combinational therapy that target multiple drug targets have become an urgent need in the field of OC treatment.

Human epidermal growth factor receptor 2 (HER2), a member of the ErbB family, is highly expressed in various tumors [12–14]. HER2 overexpression is mainly attributed to HER2 gene amplification and/or mutation, leading to the activation of signal pathways such as PI3K/Akt/mTOR and Ras/Raf/MAPK, which promote tumor cell growth, differentiation, proliferation and metastasis. Consequently, HER2 has become a highly successful therapeutic target [13, 15]. Targeted therapies for HER2 in OC include small molecule inhibitors, monoclonal antibodies, and antibody-drug conjugates (ADCs). Although less specific than monoclonal antibodies, small molecule inhibitors are more effective against tumors with low HER2 extracellular domain expression. For instance, lapatinib, an orally administered small molecule inhibitor, can inhibit the activation of HER2 and the epidermal growth factor receptor (EGFR). Clinical studies have demonstrated that lapatinib exhibits good activity in HER2-positive OC patients. Both in vitro and in vivo studies have shown its ability to partially reverse multidrug resistance in OC cells [16, 17]. Irreversible HER receptor tyrosine kinase inhibitors such as afatinib and neratinib have also shown efficacy in inhibiting OC proliferation and migration, as well as improving the chemotherapeutic response of paclitaxel-resistant OC cells [18–20]. Monoclonal antibodies targeting HER2, such as trastuzumab and pertuzumab, have been used in OC-related clinical studies. However, when used as monotherapy, these drugs have shown low response rates [21]. In a phase II clinical trial, Bookman et al. reported a response rate of 7.3% with trastuzumab in HER2 2+ and 3+ OC patients, and 39% of patients showed long-term disease stability [22]. Another phase II clinical trial by Gordon et al. showed that 4.3% of patients achieved relief with pertuzumab as monotherapy, and an additional 6.8% of patients had stable disease for at least 6 months [23].

Of particular interest, ADCs, also known as “biomissiles”, combine the specificity of monoclonal antibodies with the potency of highly cytotoxic agents, potentially reducing the severity of side effects. ADCs have recently emerged as a popular research field for anti-tumor agents [24, 25]. Successful examples of ADCs include T-DM1, T-DXd, and RC48. T-DM1, a first-in-class ADC composed of trastuzumab conjugated via a non-cleavable linker to the tubulin inhibitor DM1, has been approved for the treatment of HER2-positive metastatic breast cancer patients who have previously received trastuzumab and taxane therapy, either alone or in combination [13, 26, 27]. Preclinical studies have demonstrated that T-DM1 exhibits superior antitumor effects compared to trastuzumab or pertuzumab alone or in combination for epithelial OC [28]. Additionally, clinical studies have shown that T-DM1 achieves progression-free disease for more than 6 months in three OC patients [29]. Another ADC, T-DXd, consists of trastuzumab, a cleavable linker, and the cytotoxic topoisomerase I inhibitor deruxtecan. It is indicated for the treatment of advanced or metastatic HER2-positive breast carcinoma and low HER2-expression tumors [13, 30, 31]. RC48, an ADC composed of a humanized anti-HER2 monoclonal antibody (hertuzumab) conjugated via a cleavable linker to monomethyl auristatin E (MMAE), has been approved for the treatment of patients with locally advanced or metastatic gastric cancer and urothelial cancer [32–34]. Jiang et al. reported that RC48 alone effectively inhibits OC tumor growth in cell lines and CDX models [35]. Thus, the therapeutic strategies involving ADCs targeting HER2 for the treatment of OC remain an area that needs further exploration. The success of T-DM1, T-DXd, and RC48 in other cancers raises the possibility of their potential application in OC. Further research and clinical studies are necessary to fully understand the therapeutic potential of these ADCs in OC treatment.

Vascular endothelial growth factor receptors (VEGFRs), including VEGFR-1, VEGFR-2, and VEGFR-3, are tyrosine kinase receptors [36, 37]. VEGFRs play a crucial role in endothelial cell proliferation and migration, vascular permeability, and tumor angiogenesis by activating downstream signaling pathways such as PI3K/Akt, p38/MAPK, and PLCγ/MAPK [38, 39]. Inhibition of VEGFR signaling has emerged as an attractive therapeutic strategy in OC [40, 41], resulting in the development of cediranib (AstraZeneca), a potent oral small-molecule inhibitor of VEGFRs. Cediranib has demonstrated promising anti-tumor activity in various cancers, including colorectal cancer, lung cancer, breast cancer, glioblastoma, prostate cancer, soft tissue sarcoma, and OC [42]. However, clinical trials have shown limited overall survival benefits with the use of VEGFR inhibitors, including cediranib and bevacizumab, for the treatment of OC [43–45]. Encouragingly, the combination of cediranib and the PARP inhibitor olaparib has shown improved clinical benefits in patients with advanced OC [46–49]. This suggests that combination therapy may be a promising approach to enhance the anti-OC effects of VEGFR inhibitors.

ErbB (class I RTK) and VEGFR (class V RTK) are subfamilies of receptor tyrosine kinases that play crucial roles in controlling cell behavior and drug resistance. Inhibitors targeting these kinases have shown significant clinical benefits in cancer management [50, 51]. However, drug resistance remains a major challenge for targeted therapies, and combination treatments that target multiple pathways simultaneously have been explored as strategies to overcome this issue [52]. Both preclinical and clinical studies have provided evidence supporting the combination of agents targeting both the HER-2 and VEGFR signaling pathways. For instance, Jain et al. demonstrated that the combination of HER2 inhibitors (trastuzumab or lapatinib) with an anti-VEGFR2 antibody (bevacizumab) significantly slowed tumor growth and improved survival in patients with HER2-amplified breast cancer brain metastases [53]. Additionally, the combination of bevacizumab, trastuzumab, and capecitabine as a first-line treatment for HER2-positive locally recurrent or metastatic breast cancer resulted in an overall response rate of 73% [54]. However, there are currently a lack of reports on the dual targeting of HER2 and VEGFR in the treatment of advanced OC.

In this study, our aim is to investigate the therapeutic efficacy and underlying molecular mechanisms of RC48, an ADC targeting HER2, either alone or in combination with the VEGFR inhibitor Cediranib Maleate (CM), in both in vitro and clinically-relevant in vivo models of advanced OC. Collectively, these findings provide a preclinical foundation supporting the potential of dual targeting HER2 and VEGFR as a promising therapeutic strategy to improve outcomes in patients with OC.

## Materials and methods

### Chemicals and drugs

RC48 was purchased from RemeGenCo.Ltd. (Shandong, China), T-DXd was purchased from Daiichi SankyoCo.Ltd. (Tokyo, Japan), T-DM1 and Trastuzumab were purchased from RocheCo.Ltd. (Basel, Switzerland). VEGFR inhibitors such as Cediranib Maleate (CM), AKT activator SC79, and AKT inhibitor Akti-1/2 were purchased from Selleck (Houston, USA).

### Cell lines

Human OC cell lines A2780, OVCAR-3, SK-OV-3, HO-8910PM, and UM-UC-3 (Human Bladder Urothelial Carcinoma) were purchased from the Cell Bank of Chinese Academy of Medical Sciences (Beijing, China), Procell Life Science&Technology Co., Ltd. (Wuhan, China), and iCell Bioscience Inc. (Shanghai, China), respectively. All cell identities were validated by short tandem repeats (STR) and confirmed to be free of mycoplasma contamination. A2780, OVCAR-3 and HO-8910PM were cultured in RPMI 1640 medium (Gibco, Life Technologies) supplemented with 10% fetal bovine serum (FBS, Gibco, Life Technologies) and 1% penicillin/streptomycin (Biochem, Shenzhen, China). SK-OV-3 cells were cultured in McCoy’s 5A medium (Procell, Wuhan, China), supplemented with 10% fetal bovine serum (FBS, Gibco, Life Technologies) and 1% penicillin/streptomycin (Biochem, Shenzhen, China). UM-UC-3 cells were cultured in MEM medium (Gibco, Life Technologies), supplemented with 10% fetal bovine serum (FBS, Gibco, Life Technologies) and 1% penicillin/streptomycin (Biochem, Shenzhen, China). The cells were maintained in a humidified incubator (Thermo Fisher Scientific, Waltham) with 5% CO_2_ at 37 ℃.

### In vitro cytotoxicity assay

A2780, OVCAR-3, SK-OV-3, HO-8910PM, and UM-UC-3 cells were seeded at a density of 4000 cells/well in 96-well plates for 72 h and treated with RC48, CM, Trastuzumab, T-DM1 and T-DXd, respectively. Primary cells derived from OC PDX models were cultured in 96-well culture plates (4000 cells/well) with RC48, CM or their combination for 5 days. Cell viability was assessed using the cell Titer-Glo® assay kit (G7572, Promega, Madison, USA) following the manufacturer’s instruction, and luminescence was measured with the SPARK Multiplate Reader (TECAN, Mannedorf, Switzerland). The 50% inhibitory concentration (IC50) was calculated using non-linear regression analysis.

### IncuCyte S3 Live Cell Imaging system assay

OVCAR-3, A2780, SK-OV-3 and HO-8910PM cells were seeded in 96-well plates (4000 cells per well) for each cell line. After continuous treatment with RC48, CM, Trastuzumab, and RC48/CM combination for 60 h, cell proliferation was monitored in real-time and cell confluence curves were automatically generated using the IncuCyte® live cell analysis system (Sartorius, Gorteen, Germany).

### Western blot assay

OVCAR-3 and A2780 cells or tissue samples were lysed using SDS lysis buffer (Beyotime Biotechnology, Shanghai, China) supplemented with PMSF (Solarbio, Beijing, China) and Phosphatase Inhibitor Cocktail (Beyotime Biotechnology, Shanghai, China). The proteins were diluted in the loading buffer (Solarbio, Beijing, China), separated by SDS-PAGE, and then transferred onto PVDF membranes (Millipore, Darmstadt, Germany). Membranes were incubated with the primary antibody overnight at 4 °C and then with secondary antibodies specific to primary antibody (Supplementary Table 1). The protein band was detected by ECL reagent (Cytiva, USA). Images were taken using a Bio-Rad Multifunctional chemiluminescence imaging system.

### EdU staining assay

OVCAR-3, A2780, SK-OV-3 and HO-8910PM cells (2 × 10^5^ cells/well) were seeded on coverslips in 12-well plates and treated with RC48, CM or their combination for 24 h. After treatments, EdU staining was performed using BeyoClick™ EdU Cell Proliferation Kit (Beyotime, Shanghai, China) in accordance with the accompanying instructions. Finally, images were captured using a laser scanning confocal microscope (Zeiss880, Jena, Germany).

### Immunofluorescence

OVCAR-3, A2780, SK-OV-3, HO-8910PM, and UM-UC-3 cells (1 × 10^5^ Cells/well) were seeded on coverslips in 12-well plates for 24 h. Then they were fixed with 4% paraformaldehyde for 30 min and permeabilized with 0.1% Triton X-100 for 15 min. Then they were blocked with 1% BSA for 1 h and incubated with HER2, VEGFR2 and VEGFR3 primary antibodies (Supplementary Table 2) at 4 °C overnight. Cells were then incubated with fluorescent secondary antibodies for 1 h at room temperature. The cell nuclei were stained with DAPI. Images were captured using a laser scanning confocal microscope (Zeiss880, Jena, Germany).

### Colony formation assay

OVCAR-3, A2780, SK-OV-3 and HO-8910PM cells were seeded in 6-well plate at the density of 4,000 cells/well to adhere overnight and treated with RC48, CM or their combination for 10 days. During the drug treatment, the cell medium containing the drug was replaced every 3 days. Subsequently, colonies were fixed with 4% paraformaldehyde for 30 min and stained with 0.01% (w/v) crystal violet for 15 min at room temperature. The images of stained colonies were scanned, and the total colony area was quantified using Image J software.

### Cell migration and invasion assay

A total of 2 × 10^4^ cells of A2780 and OVCAR-3, suspended in serum-free RPMI-1640 (100 μL), were added to the top chamber of a 24-well Trans well plate (Corning, New York, USA). The cells were then treated with RC48, CM, or a combination of both for 48 h, respectively. In the lower chamber, 500 μL of medium containing 10% fetal bovine serum (FBS) was added. For invasion assays, the upper chamber membranes were coated with Matrigel (Corning, New York, USA). Subsequently, 500 μL of RPMI-1640 with 10% FBS was placed in the lower chamber, and the cells were incubated in a 5% CO_2_ environment at 37 °C for 48 h. After incubation, the cells were fixed with 4% paraformaldehyde for 30 min and stained with 0.01% (w/v) crystal violet for 15 min at room temperature. The images were then quantified using Image J software.

### RNA-Seq analysis

RNA samples were isolated from Human OC cell lines OVCAR-3 and A2780 treated with Vehicle (VEH, *n* = 3), RC48 (*n* = 3), Cediranib Maleate (CM; *n* = 3), RC48/CM (COM; *n* = 3). Total RNA was used as the input material for the RNA sample preparations. Initially, mRNA was purified from total RNA by using poly-T oligo-attached magnetic beads. To select cDNA fragments of preferentially 370 to 420 bp in length, the library fragments were purified with AMPure XP system (Beckman Coulter, Beverly, USA). After PCR amplification, the PCR product was purified by AMPure XP beads, and the library was finally obtained. Following the construction of the library, it was initially quantified using a Qubit2.0 Fluorometer, then diluted to a concentration of 1.5 ng/μL. The insert size of the library was assessed using the Agilent 2100 bioanalyzer. After insert size meets the expectation, qRT-PCR is used to accurately quantify the effective concentration of the library (the effective concentration of the library is higher than that of 1.5 nM) to ensure the quality of the library. After qualified library check, different libraries are sequenced by Illumina NovaSeq 6000 after pooling according to the requirements of effective concentration and target data volume, and 150 bp paired end reading is generated. The basic principle of Sequencing is Sequencing by Synthesis.

Image data from high-throughput sequencers were converted into sequence data (reads) by CASAVA base recognition. Raw data (raw reads) of fastq format were firstly processed through in- house perl scripts. These include the removal of reads with adapter, reads containing N base and low-quality reads. At the same time, Q20, Q30 and GC contents were calculated for clean data. All subsequent analyses were high-quality analyses based on clean data. Index of the reference genome was built using Hisat2 (v2.0.5) and paired-end clean reads were aligned to the reference genome using Hisat2 (v2.0.5) after reference genome and gene model annotation files were downloaded from genome website.

FeatureCounts (1.5.0-p3) was used to calculate the reads numbers mapped to each gene. The FPKM of each gene was then calculated based on the length of the underlying cause, and the readings mapped to that gene were calculated. Differential expression analysis was performed using the DESeq2 R package ( 1.20.0). The resulting *P*-values were adjusted using the Benjamini and Hochberg’s approach. padj < = 0.05 and |log2(foldchange)|> = 1 were set as the threshold for significantly differential expression.

Based on the condition that the *P* value < 0.05, we used clusterProfiler R package (3.8.1) to test the statistical enrichment of differential expression genes in KEGG pathways in accordance with the KEGG database (http://www.genome.jp/kegg/). The gene set enrichment analysis (GSEA) was performed using the Molecular Signatures Database (MSigDB) hallmark gene set collection. The GSEA Analysis tool is available at http://www.broadinstitute.org/gsea/index.jsp.

### Analysis of apoptosis via flow cytometry

A2780 and OVCAR-3 cells (1 × 10^6^/well) were cultured in 6-well plates and treated with RC48, CM or their combination for 48 h. Flow cytometry was performed using the Annexin V-propidium iodide (PI) apoptosis detection kit (Beyotime Biotechnology, Shanghai, China) on a BD FACSCantoII instrument (USA) to determine cell apoptosis.

### In vivo therapeutic efficacy

BALB/c nude mice (5–6 weeks old) were purchased from SJA Laboratory Animal Co., Ltd. (Hunan, China) for CDXs. NOD/SCID mice, 5–6-week-old female, were provided by Cyagen Biosciences Inc (Suzhou, China) for PDXs. All mice were fed standard laboratory chow and provided ad libitum access to water under specific pathogen-free (SPF) conditions. All animal experiments were approved and performed in full compliance with the guidelines approved by the Biomedical Research Ethics Committee, Gannan Medical University (Jiangxi, China). For the CDX models, 3 × 10^6^ A2780 and OVCAR-3 cells were suspended in 100 μL of PBS and injected subcutaneously into the right flank of the experimental mice. For the PDX models, the mice were subcutaneously inoculated in the right flank with OCtissue fragments measuring 2 to 3 mm^3^. Once the tumor mean volume reached 100–150 mm^3^, the mice were randomized into 4 groups (*n* = 3–5/group) and injected intravenously with saline, 5 mg/kg of RC48, 10 mg/kg of RC48, and 10 mg/kg of Trastuzumab once weekly. In addition, for the combination therapy study, the mice were treated with saline, 2 mg/kg RC48 for QW × 3, 0.5 mg/kg CM for QD × 22, and a combination of RC48 and CM. The tumor sizes and body weight of the mice were measured twice a week using a caliper, and the tumor volumes were calculated using the formula: tumor volume (mm^3^) = length × (width)^2^ × 0.5. The inhibition rate of the tumor growth (TGI) was calculated as (1-treated tumor volume/control tumor volume) × 100%. If the tumor volume did not exceed 2000 mm^3^ by the end of the experiment, the mice were euthanized, and organs were collected for subsequent analyses.

### TUNEL assay

The TdT-UTP nick end-labeling (TUNEL) assay was performed according to the protocol of the One Step TUNEL Apoptosis Assay Kit (C1088, Beyotime, Shanghai, China). Briefly, tumor tissue sections were fixed with 4% paraformaldehyde for 30 min and permeabilized with 0.1% Triton X-100 for 10 min. Slides were incubated with the TUNEL detection reagent for 60 min at 37 °C. Tissue samples were observed under a laser scanning confocal microscope (Zeiss880, Jena, Germany).

### H&E staining and immunohistochemistry

Ninety epithelial OC (EOC) tissues including High-grade serous adenocarcinoma, low-grade serous adenocarcinoma, clear cell adenocarcinoma, mucinous cystadenocarcinoma, endometrioid adenocarcinoma and carcinosarcoma, were obtained from the Department of Pathology, First Affiliated Hospital, Gannan Medical University (Supplementary Tables 3 and 4). All tissues were examined by specialists using the World Health Organization criteria. Determination of OC stage and grade was according to the International Federation of Gynecology and obstetrics. The use of these specimens and patient’s information was approved by the Ethics Committee of First Affiliated Hospital, Gannan Medical University. The OC CDXs and PDXs xenograft tumor tissues were fixed with formalin and Paraffin-embedded. H&E staining was performed using a standard histological protocol. OC tissues were collected and made into 4-μm-thick paraffin tissue microarrays for immunohistochemistry (IHC) staining. The staining procedure was carried out using the UltraSensitive™ SP (mouse/rabbit) IHC Kit and DAB Kit (MXB, Fuzhou, China) according to the standard procedure. Imaging was captured using the TissueFAXS Plus system (version 7.0, TissueGnostics GmbH, Vienna, Austria).

### Statistical analysis

All experimental data were presented as the mean ± standard error of mean (SEM) of 3 independent experiments, using GraphPad Prism 5 software. IC50 values were determined through nonlinear regression analysis of concentration-response curves, utilizing SPSS 16.0. The statistical significance between the two groups was analyzed by one-way analysis of variance (ANOVA) or Student’s *t*-test when compared with the untreated or vehicle group. For all experiments, differences were considered significant at *P* < 0.05. *0.01 ≤ *P* < 0.05; **0.001 ≤ *P* < 0.01; ***P < 0.001.

## Results

### HER2 and VEGFR were overexpressed in OC cell lines and tumor tissues

**Fig. 1:**
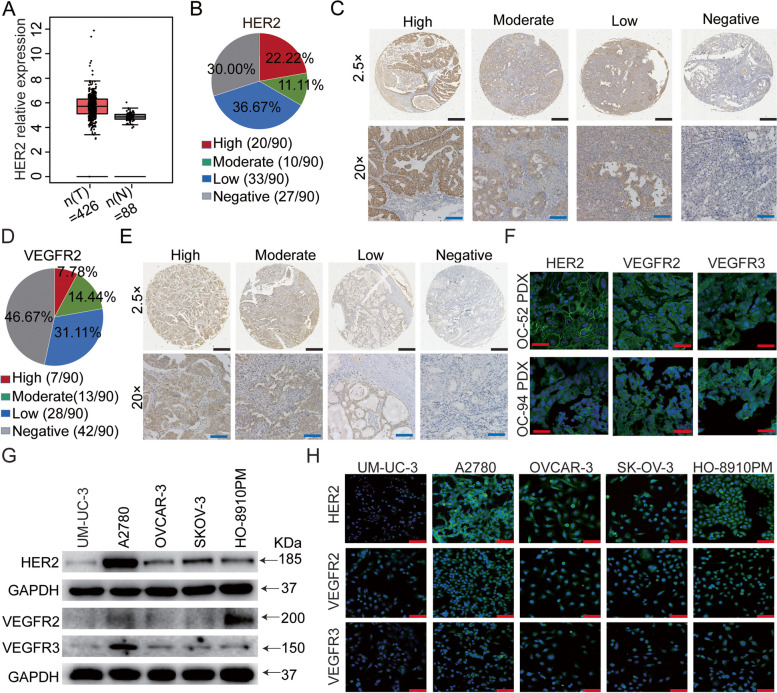
HER2 and VEGFR were overexpressed in OC. **A** Box plot downloaded from the GEPIA database shows high expression of HER2 in OC tissues. **B**, **D** The percentage of HER2 and VEGFR2 expression in 90 OC tumors, categorized into four staining intensities: high, moderate, low, and negative. **C**, **E** Representative images of 4 different staining intensities of HER2 and VEGFR2 immunohistochemistry (IHC) on human OC tumor tissue microarrays (TMA). Images were captured at 25 × magnification (upper panels) and 200 × magnification (lower panels), with scale bars = 200 µm (upper panels) and scale bars = 50 µm (lower panels), respectively. **F** Immunofluorescence was used to analyze the expression of HER2 and VEGFR in two OC PDX tissues. **G**, **H** The protein expression levels of HER2 and VEGFR in four OC cell lines and UM-UC-3 cell lines through western blotting (**G**) and immunofluorescence (**H**) assay. Immunofluorescence images (**F**, **H**) were captured at 400 × magnification, with scale bars = 20 µm

To assess the expression of HER2 and VEGFR in OC, we analyzed the GEPIA database and performed immunohistochemistry assays on a tissue microarray containing 90 cores of human OC tumor specimens. Our analysis revealed that HER2 expression was significantly upregulated in OC tissues compared to normal ovarian tissues (Fig. 1A). This finding was consistent with previous reports [55, 56]. HER2 was overexpressed in OC specimens but rarely positive in normal ovarian tissues. Immunohistochemistry assays showed that HER2 and VEGFR2 were positive in 70% (63/90) and 53.3% (42/90) of the 90 OC patient specimens, respectively. Staining intensities for HER2 and VEGFR2 ranged from high to negative in different proportions of the specimens (Fig. 1B-E). We also observed positive expression of HER2, VEGFR2, and VEGFR3 in two OC PDX models (Fig. 1F).

To select appropriate OC cell lines for further in vitro and in vivo evaluation, we examined the expression of HER2, VEGFR2, and VEGFR3 in four OC cell lines and one bladder cancer cell line using Western blot and immunofluorescence assays. Our data showed that A2780 cells exhibited higher expression levels of HER2, VEGFR2, and VEGFR3, while OVCAR-3, SK-OV-3, and HO-8910PM cells showed moderate expression levels. On the other hand, UM-UC-3 cells exhibited lower expression levels of HER2, VEGFR2, and VEGFR3 (Fig. 1G). Immunofluorescence assays confirmed similar expression levels of HER2, VEGFR2, and VEGFR3 in A2780, OVCAR-3, SK-OV-3, and HO-8910PM cells, while UM-UC-3 cells showed lower expression levels, consistent with the Western blot results (Fig. 1H).

In summary, our findings indicate that HER2, VEGFR2, and VEGFR3 are overexpressed in OC tumors. These expression patterns provide a basis for further investigation of the therapeutic potential of targeting HER2 and VEGFR in OC.

### RC48 and CM alone remarkably inhibit the proliferation of OC cells in vitro

**Fig. 2:**
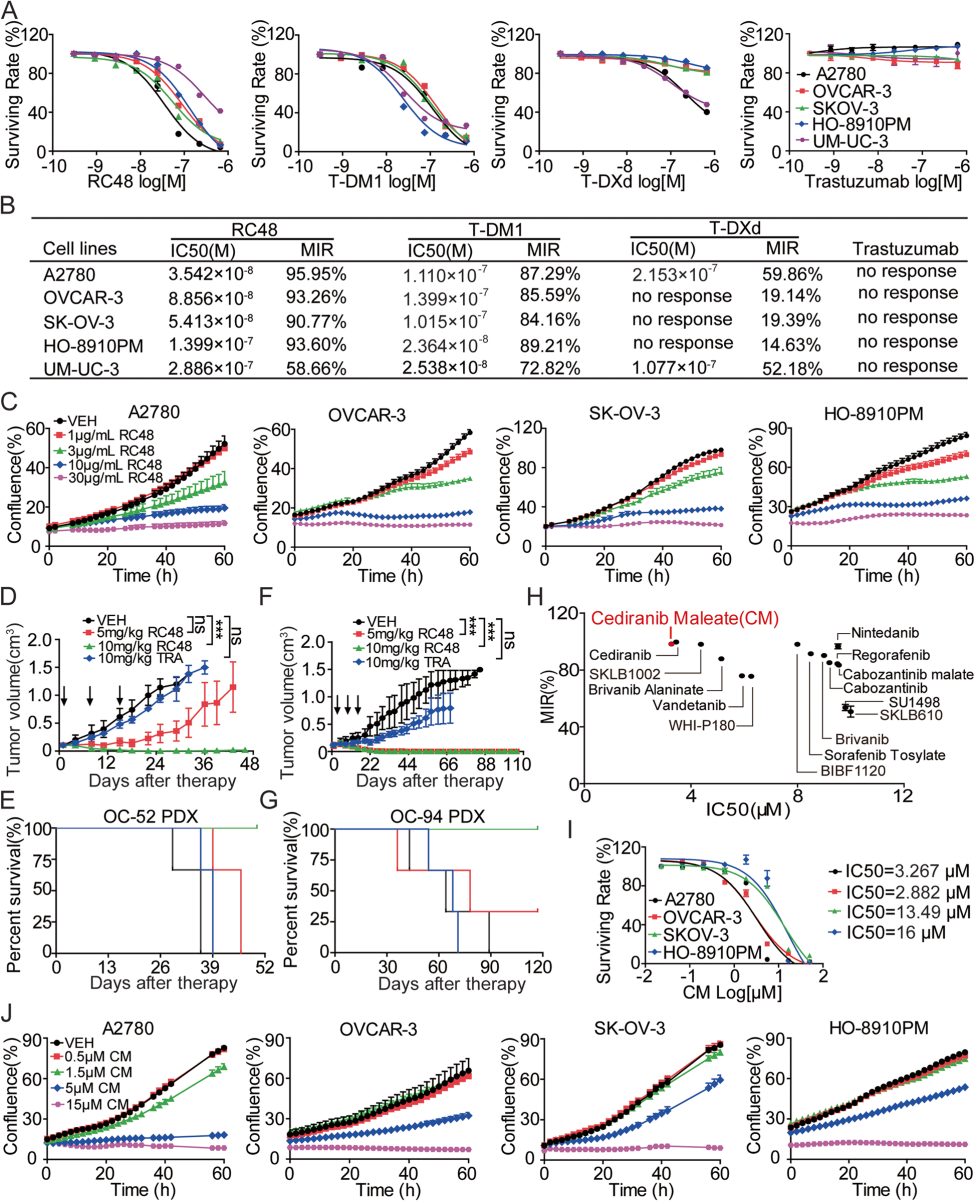
Antiproliferative effects of RC48 and CM alone on OC cells. **A**, **B** A2780, OVCAR-3, SK-OV-3, HO-8910PM and UM-UC-3 cells were treated with RC48, T-DM1, T-DXd and trastuzumab for 72 h, respectively. Cell viability was detected using Cell Titer-Glo cytotoxicity assays. **C** The proliferation of OC cells treated with RC48 for 60 h was observed using the Incucyte real-time cell analysis system. **D**-**G** The anti-tumor activity and survival benefit of 5 and 10 mg/kg RC48 were evaluated in the OC-52 (**D**, **E**) and OC-94 (**F**, **G**) PDX models. **H** The toxic effects of VEGFR inhibitors on A2780 cells were evaluated, and a scatter map of MIR-IC50 was generated. **I** A2780, OVCAR-3, SKOV-3 and HO-8910PM cells were treated with CM for 72 h, and cell viability was detected using Cell Titer-Glo cytotoxicity assays. **J** The proliferation of OC cells treated with CM for 60 h was observed using the Incucyte real-time cell analysis system. Data represent the mean ± standard error of the mean (SEM) of at least three independent experiments, and statistical significance was assessed by unpaired T-test (***p < 0.001)

To investigate the effects of RC48, T-DM1, T-DXd, and trastuzumab on OC cell survival in vitro, we treated A2780, OVCAR-3, SK-OV-3, HO-8910PM, and UM-UC-3 cells with these agents and assessed cell viability using the Cell Titer-Glo Cell Viability assay. Our results showed that both RC48 and T-DM1 significantly inhibited the growth of the four OC cell lines in a dose-dependent manner. The IC50 values of RC48 for A2780, OVCAR-3, SK-OV-3, and HO-8910PM cells were 35.42 nM (MIR = 95.95%), 88.56 nM (MIR = 93.26%), 54.13 nM (MIR = 90.77%), and 116.4 nM (MIR = 93.60%), respectively. However, RC48 had the weakest inhibitory effect on UM-UC-3 cells, with an IC50 value of 123.8 nM and MIR of only 54.85% (Fig. 2A and B). T-DM1 displayed moderate antiproliferative activity in A2780, OVCAR-3, SK-OV-3, HO-8910PM, and UM-UC-3 cells, with IC50 values of 111.0 nM (MIR = 87.29%), 139.9 nM (MIR = 85.59%), 101.5 nM (MIR = 84.16%), 23.64 nM (MIR = 89.21%), and 25.38 nM (MIR = 72.82%), respectively. T-DXd showed moderate antiproliferative activity only in A2780 and UM-UC-3 cells. Trastuzumab showed no cytotoxicity in these cell lines (Fig. 2A and B).

We further evaluated the effects of RC48, T-DM1, T-DXd, and trastuzumab on cell confluence using IncuCyte Live Cell Imaging experiments in A2780, OVCAR-3, SK-OV-3, and HO-8910PM cells. Similar inhibitory responses were observed in all four cell lines (Fig. 2C; Supplementary Figs. 1 and 2), confirming the antiproliferative effects of these agents. In addition, EdU assays and clonogenic assays confirmed that RC48 significantly and dose-dependently inhibited cell proliferation in A2780, OVCAR-3, SK-OV-3, and HO-8910PM cells (Supplemental Figs. 4 and 5). Therefore, RC48 monotherapy demonstrated excellent antiproliferative activity in vitro.

To evaluate the therapeutic efficacy of RC48 in vivo, we used PDX models, which maintain the key pathological and genetic characteristics of the original tumors and are considered a successful tool for preclinical drug development [57, 58]. Treatment with RC48 resulted in significant and dose-dependent tumor regression in two OC PDX models compared to the vehicle and 10 mg/kg trastuzumab groups. Furthermore, RC48 treatment led to a significant improvement in overall survival compared to their respective VEH groups (Fig. 2D-G). Notably, the therapeutic efficacy of RC48 resulted in tumor remission without relapse until the end of the 118-day study period in the OC-94 PDX model. Importantly, none of the treatments caused significant changes in mouse body weight (Supplementary Fig. 6).

To explore the therapeutic efficacy of VEGFR inhibitors, we screened 22 VEGFR inhibitors in A2780 cells using Cell Titer-Glo cytotoxicity assays. Our data demonstrated that CM exhibited stronger antiproliferative activity compared to the other VEGFR inhibitors (Fig. 2H and Supplementary Fig. 3). We further confirmed the inhibitory effects of CM on cell viability in A2780, OVCAR-3, SK-OV-3, and HO-8910PM cell lines using Cell Titer-Glo cytotoxicity assays, IncuCyte Live Cell Imaging experiments, EdU assays, and clonogenic assays (Fig. 2I, J and Supplementary Figs. 2, 4, and 5). The IC50 values of CM for A2780, OVCAR-3, SK-OV-3, and HO-8910PM cells were 3.267 μM, 2.882 μM, 13.49 μM, and 16.00 μM, respectively.

In conclusion, both RC48 and CM alone revealed excellent anti-tumor activity in these OC cells, suggesting their potential as anti-cancer agents for the treatment of OC.

### RC48 and CM cooperatively inhibit the proliferation and motility of OC cells

**Fig. 3:**
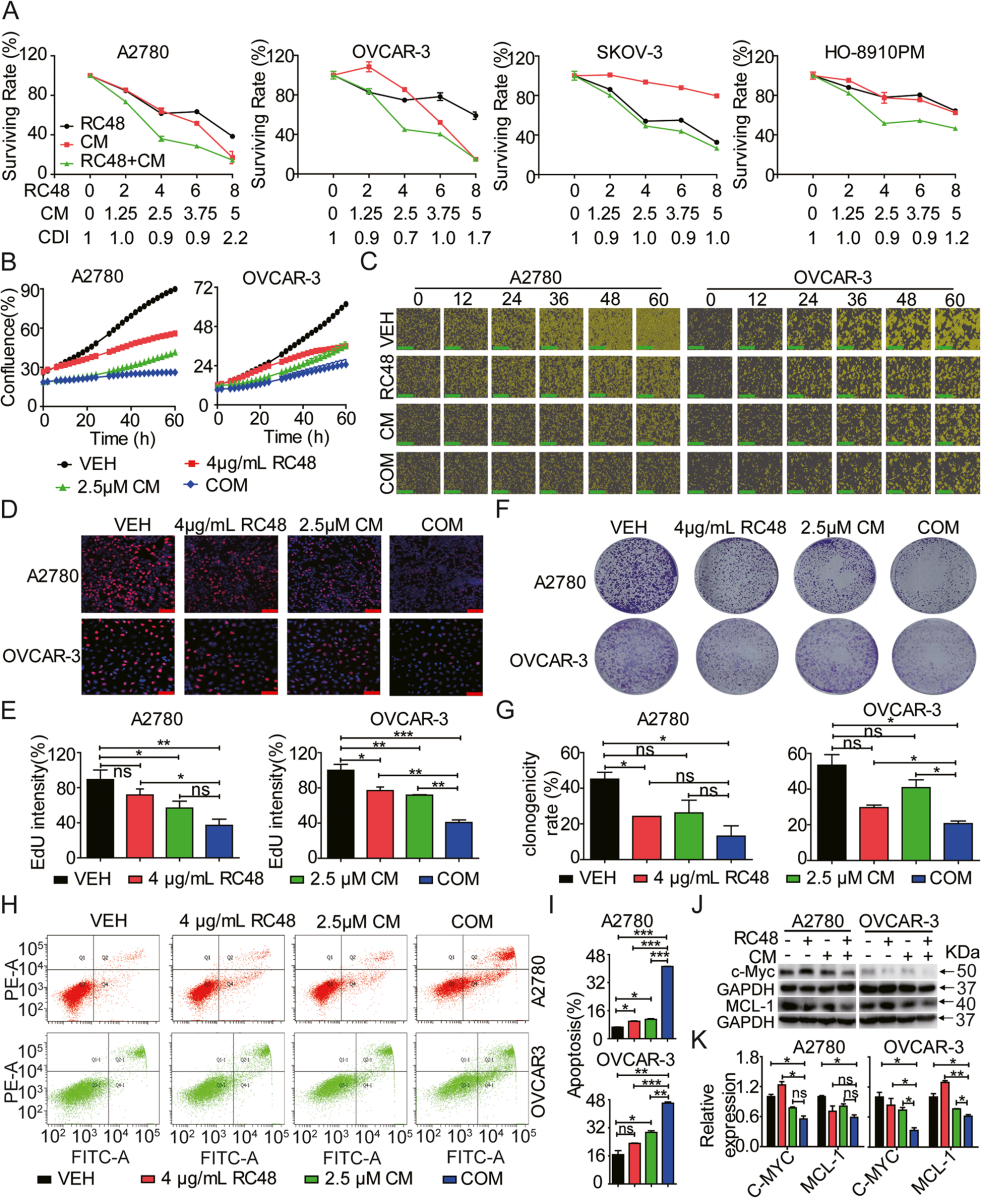
Synergistic inhibition of OC Cell Proliferation in vitro by RC48 and CM. **A** A2780, OVCAR-3, SK-OV-3 and HO-8910PM cells were treated with various concentrations of RC48, CM, or their combination (COM) for 72 h. Cell viability was detected using Cell Titer-Glo cytotoxicity assays. **B**, **C** The proliferation of A2780, OVCAR-3 cells treated with vehicle control (VEH), 4 μg/mL RC48, 2.5 μM CM, or COM for 60 h was observed using the Incucyte real-time cell analysis system. Images captured at 100 × magnification, respectively. Scale bars = 400 µm. **D**, **E** A2780 and OVCAR-3 cells were treated with 4 μg/mL RC48, 2.5 μM CM, or COM for 24 h, and cell proliferation was determined using the EdU assay. Images were captured using a laser scanning confocal microscope. Images were captured at 100 × magnification (**C**) and 400 × magnification (**D**), with scale bars = 400 µm and scale bars = 20 µm, respectively. **F**, **G** The anti-proliferative effects were determined by assessing the area of colonies stained with crystal violet. **H**, **I** A2780 and OVCAR-3 cells were treated with 4 μg/mL RC48, 2.5 μM CM, or COM for 48 h. Apoptosis was analyzed by staining the cells with Annexin V-FITC/PI and flow cytometry. **J**, **K** Western blotting was performed to detect c-Myc, and MCL-1 levels as indicators of apoptotic cell death. GAPDH was used as loading control. The experiments were conducted in triplicate. Data represent the mean ± SEM of three independent experiments. Statistical significance was assessed using one-way ANOVA with Bonferroni post hoc test (*p < 0.05; **p < 0.01; ***p < 0.001)

We investigated the synergistic effects of combining RC48 and CM on OC cells. Cell viability was assessed using the Cell TiterGlo Cell Viability assay in A2780, OVCAR-3, SK-OV-3, and HO-8910PM cells treated with RC48 alone or in combination with CM. Various combination doses showed synergistic effects in A2780, OVCAR-3, SK-OV-3, and HO-8910PM cells. Among them, the combination treatment of 4 μg/mL RC48 and 2.5 μM CM exhibited the most superior synergistic effect in these cell lines (Fig. 3A). Notably, the combination treatment showed greater inhibition in A2780 and OVCAR-3 cells compared to SK-OV-3 and HO-8910PM cells. Therefore, we selected A2780 and OVCAR-3 cells treated with RC48 alone or in combination with CM for further in vitro and in vivo experiments.

Cell survival and cell confluency were assessed using IncuCyte Live Cell Imaging experiments. The combination of RC48 and CM synergistically and time-dependently induced cell death in A2780 and OVCAR-3 cells compared to other treatment groups (Fig. 3B, C). Additionally, EdU (Fig. 3D, E) and clonogenic (Fig. 3F, G) assays revealed that combination therapy of RC48 and CM reduced cell proliferation and colony formation in these OC cells compared to each treatment alone. Flow cytometry analysis demonstrated that the combination of RC48 and CM cooperatively increased the percentage of apoptotic cells compared to RC48 or CM alone (Fig. 3H, I). At the molecular level, western blot analysis showed that the combination treatment downregulated the expression levels of apoptosis-related proteins, such as c-Myc and MCL-1, consistent with the apoptosis data obtained by flow cytometry (Fig. 3J, K).

**Fig. 4:**
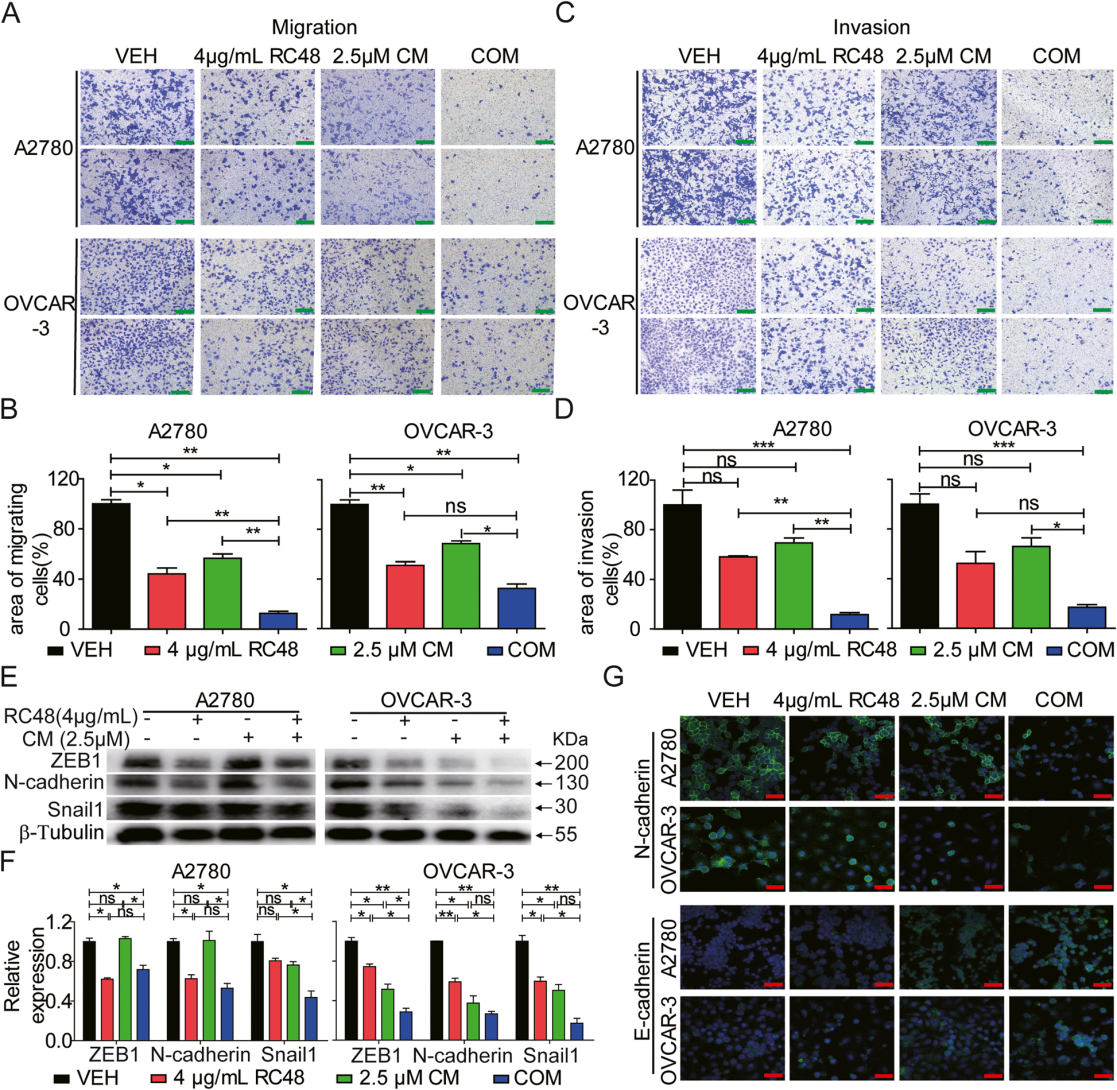
RC48 and CM cooperatively suppressed the cell motility of OC cells. **A**, **B** A2780 and OVCAR-3 cells were exposed to VEH, 4 μg/mL RC48, 2.5 μM CM, or COM for 48 h. Cell motility was detected using transwell migration (**A**, **B**) and invasion (**C**, **D**) assays. Images were captured at 100 × magnification (**A**, **B**), with scale bars = 100 µm. **E**–**F** A2780 and OVCAR-3 cells were exposed to different treatments for 60 h, including 4 μg/mL RC48, 2.5 μM CM, or COM. Western blotting was performed to examine the expression of EMT markers (ZEB1, N-cadherin, and Snai1). β-tubulin served as the loading control. **G** A2780 and OVCAR-3 cells were treated with 4 μg/mL RC48, 2.5 μM CM, or COM for 48 h. Representative immunofluorescence images of N-cadherin and E-cadherin in A2780 and OVCAR-3 cells were captured using LSCM at 400 × magnification. Scale bars = 20 µm. Nuclei were counterstained with DAPI. The data shown represent the mean ± SEM from three independent experiments performed in triplicate. Statistically significant values of *p < 0.05, **p < 0.01, and ***p < 0.001

Tumor cell dissemination is a characteristic feature of malignant tumors and a major cause of tumor recurrence and distant metastasis. Epithelial-to-mesenchymal transition (EMT) is a crucial pathway for invasion and migration of epithelial cell tumors [59, 60]. We examined the combined effect of RC48 and CM on motility in A2780 and OVCAR-3 cells. Transwell cell migration/invasion assays showed that RC48 and CM alone moderately inhibited the migration and invasion of A2780 and OVCAR-3 cells. However, the combination of RC48 and CM displayed a stronger inhibitory effect on the migration and invasion of OC cells (Fig. 4A-D). Furthermore, we assessed the expression of EMT markers, including ZEB1, N-cadherin, and Snail1. The combination of RC48 and CM decreased the protein levels of ZEB1, N-cadherin, and Snail1 compared to the single-agent treatment groups (Fig. 4E, F). Immunofluorescence assay further demonstrated that the combination of RC48 and CM downregulated the mesenchymal marker N-cadherin and upregulated the epithelial marker E-cadherin in A2780 and OVCAR-3 cells compared to other treatment groups (Fig. 4G).

Overall, these findings indicate that the combination of RC48 and CM synergistically inhibits the proliferation, migration, and invasion of OC cells in vitro.

### Combination of RC48 and CM synergistically inhibited cell growth via the PI3K-AKT pathway

**Fig. 5:**
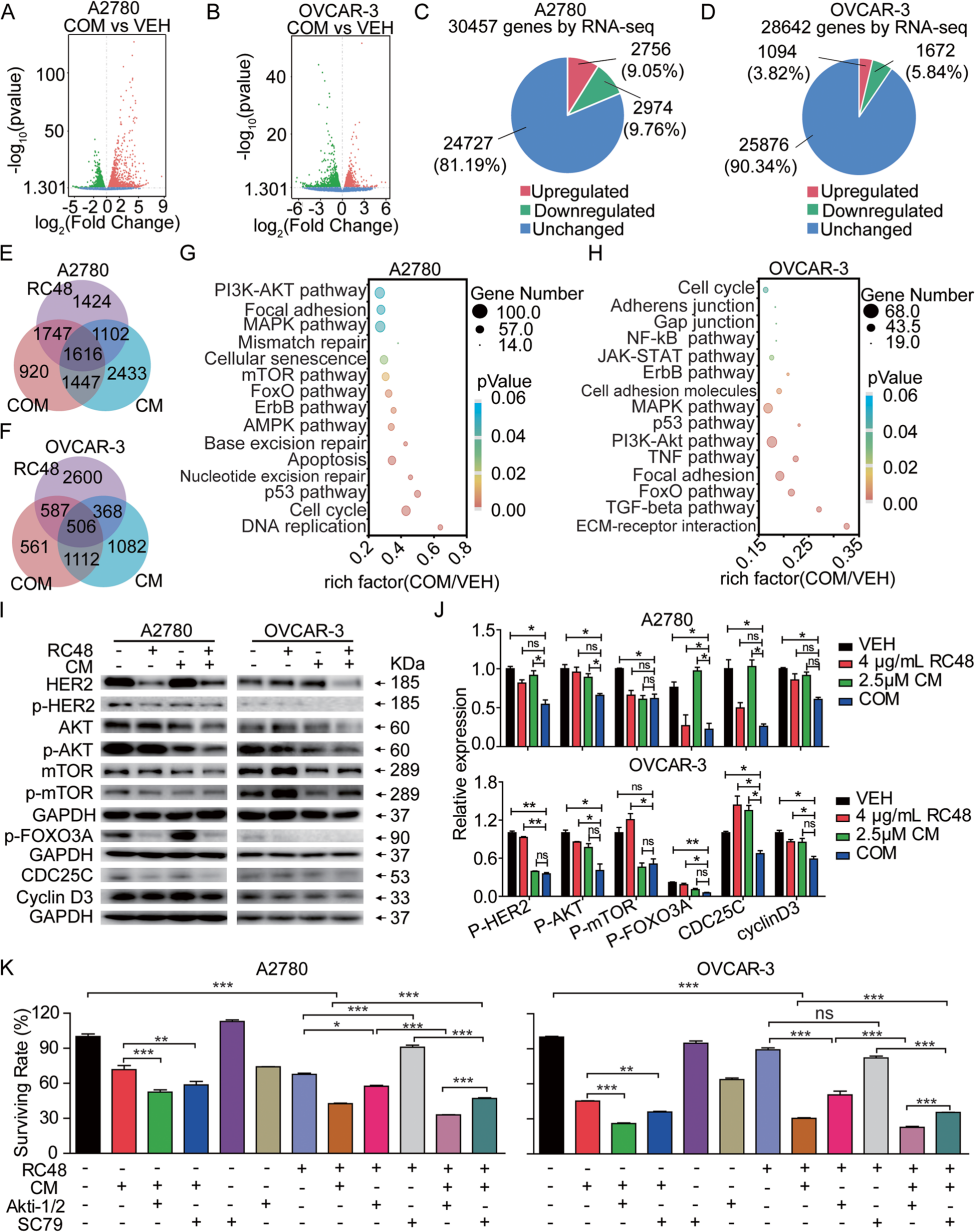
Effects of RC48 and CM combinational treatment on global gene expression in OC cells. Gene expression studies were performed using RNA-Seq in A2780 cells and OVCAR-3 cells treated with VEH, 4 μg/mL RC48, 2.5 µM CM or COM for 48 h. Each group consisted of triplicate samples. **A**, **B** The expression of DEGs in A2780 (**A**) and OVCAR-3 (**B**) cells between the VEH and COM groups is shown. Upregulated genes are depicted in red, while downregulated genes are depicted in green. Gene expression values are presented as the log10 of tag counts. **E**, **F** The number of overlapping DEGs in RC48, CM, and COM treated cells compared to VEH-treated cells is displayed. **G**, **H** Kyoto Encyclopedia of Genes and Genomes (KEGG) analysis was performed on DEGs in COM-treated samples compared to VEH-treated samples. **I**, **J** Analysis of the PI3K/AKT signaling pathway and cell cycle-related proteins. GAPDH was used as the loading control. **K** The effect of 5 µM AKTi1/2 and 5 µM SC79 on the combined effect of RC48 and CM in OC cells was assessed using Cell Titer-Glo cytotoxicity assays. Data represent the mean ± SEM of at least three independent experiments, and statistical significance was assessed using an unpaired T-test (*P < 0.05, **P < 0.01, ***p < 0.001)

To further explore the molecular mechanisms underlying the combination treatment, we performed RNA-Seq analysis in A2780 and OVCAR-3 cells treated with RC48 and CM, alone or in combination. Multidimensional scaling analysis revealed significant differential expression of genes in the COM group compared to the VEH group. To visualize the differences, we performed multidimensional scaling analysis, which demonstrated distinct clustering of the COM group compared to the VEH group (Fig. 5A-F, Supplementary Fig. 7). This clustering pattern indicated that the combination treatment induced significant changes in gene expression profiles compared to the VEH group. By using RNA-Seq analysis and multidimensional scaling, we gained insights into the global gene expression alterations induced by the combination treatment. These findings contribute to our understanding of the molecular changes associated with the therapeutic effects of RC48 and CM in OC cells. Specifically, in A2780 and OVCAR-3 cells, a substantial number of genes showed differential expression in the combination group. Out of these genes, 5730 (18.81%) and 2766 (9.66%) genes were significantly differentially expressed in A2780 and OVCAR-3 cells, respectively. Among the differentially expressed genes (DEGs), 2756 (9.05%) and 1094 (3.82%) were upregulated, while 2974 (9.76%) and 1672 (5.84%) were downregulated compared to the VEH group.

To gain further insights into the functional implications of these DEGs, we performed KEGG pathway analysis. This analysis revealed the modulation of multiple signaling pathways by the COM treatment in A2780 and OVCAR-3 cells. Specifically, the COM treatment affected pathways such as the PI3K-AKT pathway, FOXO pathway, apoptosis, cell cycle, p53 pathway, focal adhesion, and cellular senescence (Fig. 5G, H). These pathways play crucial roles in cancer cell proliferation and metastasis. Moreover, the key genes associated with these pathways were significantly altered in the COM treatment group (Supplementary Figs. 8–9, Supplementary Tables 5–14). Furthermore, protein-level analysis confirmed that RC48 plus CM downregulated key proteins involved in the PI3K-AKT pathway, FOXO pathway, and cell cycle in A2780 and OVCAR-3 cells, consistent with the RNA-Seq analysis (Fig. 5I, J). These results highlight the superior ability of the COM treatment to block key cancer-related pathways.

In order to investigate the role of the PI3K-AKT pathway in the effects of RC48 alone or in combination with CM, we assessed the impact of Akti1/2, a small molecule AKT inhibitor. The results revealed that  Akti1/2 significantly enhanced the anti-proliferative activity of RC48 alone or in combination with CM in A2780 and OVCAR-3 cells. Notably, AKTi1/2 combined with RC48 or CM exhibited stronger inhibitory effects than the monotherapy groups. Additionally, AKTi1/2 + RC48 + CM demonstrated more significant anti-proliferative activity compared to RC48 plus CM in A2780 and OVCAR-3 cells (Fig. 5K). Conversely, the AKT activator SC79 reversed the inhibitory effects of RC48 alone or in combination with CM and promoted cell survival (Fig. 5K). These results suggest that the inhibition of tumor growth by RC48 alone or in combination with CM occurs via the PI3K-AKT signaling pathway in A2780 and OVCAR-3 cells.

In conclusion, our findings indicate that combination treatment of RC48 and CM efficiently blocks key cancer-driving signaling pathways and has a broader impact on additional pathways. These findings emphasize the need for further investigation into the potential therapeutic implications of these pathways in OC treatment.

### Combination treatment of RC48 and CM affected signaling pathways and unique OC-associated prognostic genes

**Fig. 6:**
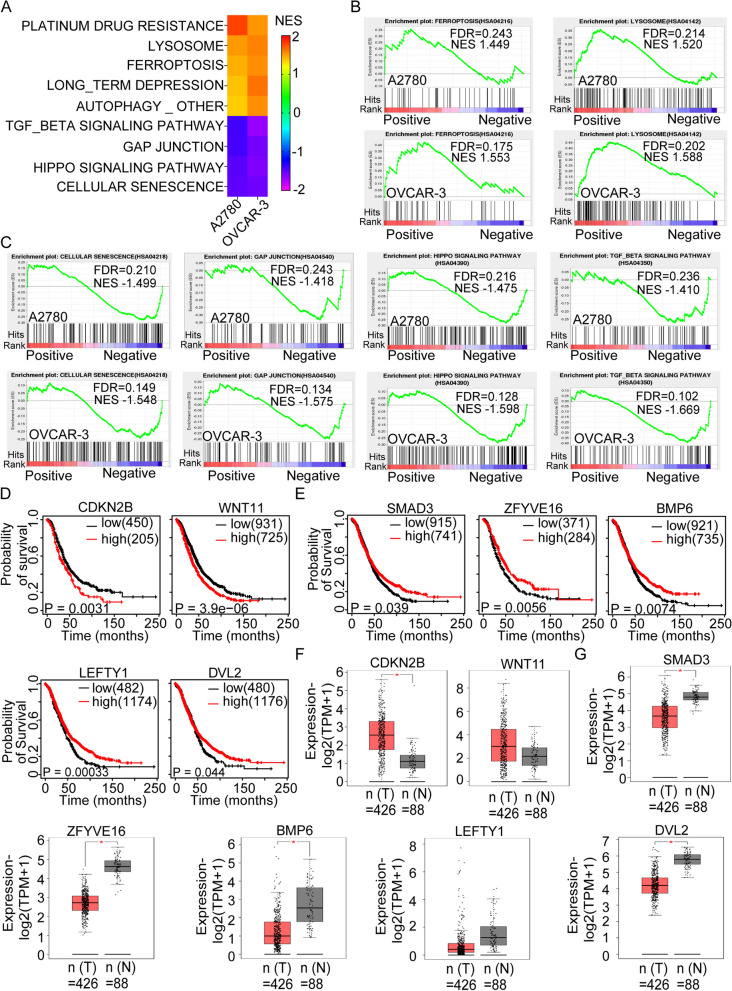
Combination treatment of RC48 and CM affected signaling pathways and unique OC-associated prognostic genes. GSEA was performed using the 50 HALLMARK gene set database in MSigDB to analyze the most common DEGs in A2780 and OVCAR-3 cells treated with the combination therapy. **A** Positively and negatively enriched gene sets responsive to RC48 plus CM were identified using GSEA. **B** GSEA revealed strong positive enrichment of cell death-related pathways, such as ferroptosis and lysosomes, in A2780 and OVCAR-3 cells under combination treatment. **C** Negative enrichment was observed in cellular senescence, GAP junction, the hippo signaling pathway, and TGF-β signaling pathway, which are related to cell growth and adhesion pathways. NES, Normalised Enrichment Score; FDR, false discovery rate. **D**, **E** The relationship between different expressed genes enriched by GSEA and overall survival rate was evaluated using Kaplan-Meier analysis. Lower expression of CDKN2B and WNT11 was found to be beneficial to the overall survival rate of patients with OC (*p* < 0.01), while high expression of SMAD3, ZFYVE16, BMP6, LEFTY1, and DVL2 significantly improved the overall survival of OC patients (*p* < 0.05). Significance was determined using the log-rank (Mantel-Cox) test, and *p* < 0.05 was considered significant. **F**, **G** GEPIA was used to analyze the differential expression of genes enriched by GSEA in OC tissues and adjacent tissues

We conducted gene set enrichment analysis (GSEA) to explore the functional and signaling pathway changes induced by the combination treatment of RC48 and CM in ovarian cancer. GSEA analysis identified negatively enriched and positively enriched gene sets in A2780 and OVCAR-3 cells after the combination treatment (Fig. 6A and Supplementary Table 15). These gene sets provide insights into the biological processes and pathways affected by the treatment.

In particular, we observed that two signaling pathways, ferroptosis and lysosomes, were significantly upregulated following the combination treatment (Fig. 6B). Conversely, four signaling pathways, including cellular senescence, GAP junction, hippo signaling pathway, and TGF-β signaling pathway, were significantly downregulated (Fig. 6C). These findings suggest that the combination treatment of RC48 and CM has a profound impact on these specific signaling pathways in OC cells.

To validate the biological relevance of the genes regulated by the combination treatment, we analyzed the relationship between the GSEA-enriched genes and overall survival using Kaplan-Meier curves in the ovarian cancer cohort from The Cancer Genome Atlas (TCGA) dataset. We found that a low expression level of CDKN2B and WNT11 was associated with longer survival in ovarian cancer patients, while high expression levels of SMAD3, ZFYVE16, BMP6, LEFTY1, and DVL2 were significantly associated with poor survival (Fig. 6D, E).

Furthermore, we investigated the expression levels of these genes in OC tissues and in the combination treatment group. We found that CDKN2B and WNT11 were significantly upregulated in OC tissues and downregulated in the combination treatment group, supporting their potential role as prognostic markers (Fig. 6F). Conversely, SMAD3, ZFYVE16, BMP6, LEFTY1, and DVL2 showed weak expression in OC tissues but were strongly expressed in the combination treatment group (Fig. 6G).

Overall, these results provide a comprehensive analysis of the functional and prognostic implications of the gene expression changes induced by the combination treatment of RC48 and CM in OC. These findings contribute to our understanding of the underlying mechanisms and potential therapeutic targets in this context.

### RC48 synergized with CM remarkably to improve the anti-tumor activity in CDX models

**Fig. 7:**
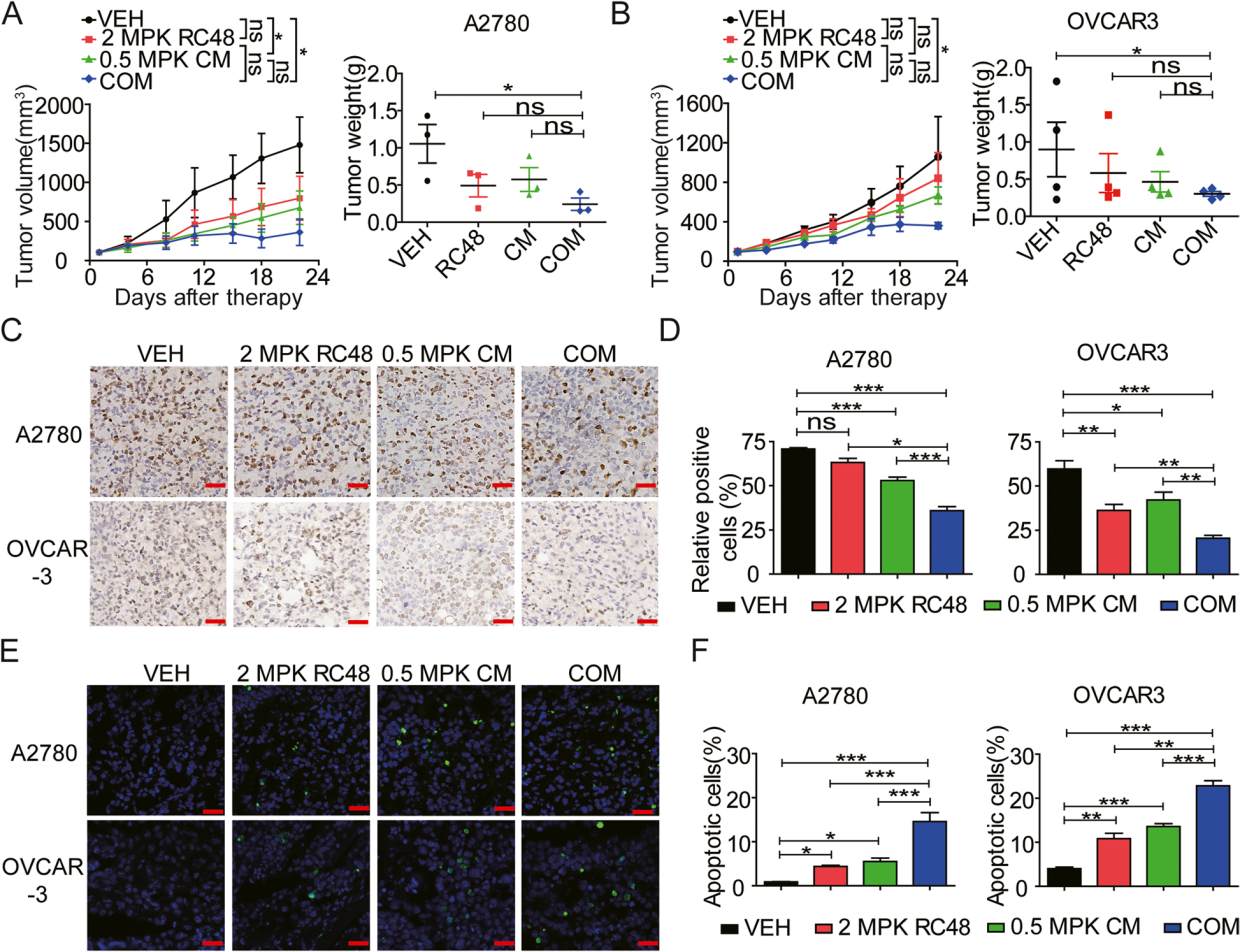
Synergistic inhibition of tumor growth in OC CDX models by RC48 and CM. In both CDX models, mice were intravenously administered with VEH, 2.0 mg/kg (MPK) RC48 (once a week for 3 times), 0.5 MPK CM (once every day), or COM. **A**, **B** Tumor growth curve and transplanted tumor weight were evaluated in the A2780 (**A**) and OVCAR-3 (**B**) CDX models. **C**, **D** Representative IHC image of Ki‐67 staining (a proliferation index) in A2780 (upper panel) and OVCAR-3 (lower panel) xenograft tumors. Images were captured using TissueFAXS Plus at 400 × magnification. Scale bars = 20 µm. **E**, **F** The quantification of apoptotic cells in A2780 (upper panel) and OVCAR-3 (lower panel) xenograft tumors was determined using TUNEL assay. Apoptotic cells are represented by green fluorescent-stained cell nuclei. Images were captured using LSCM at 400 × magnification. Scale bars = 20 µm. The data shown represent the mean ± SEM from three independent experiments performed in triplicate. All treated groups compared to VEH group or COM group compared to single agent group. Statistically significant values are denoted as *p < 0.05, **p < 0.01, and ***p < 0.001

To assess the translational potential of our in vitro findings, we investigated the synergistic effects of RC48 combined with CM in in vivo models. Subcutaneous xenografts were established using the A2780 and OVCAR-3 cell lines. Mice were then treated once a week for three weeks (QW × 3) with either vehicle (VEH), 2 mg/kg RC48, 0.5 mg/kg CM alone, or the combination treatment (COM). Tumor growth was monitored for 22 days. In the A2780 CDX model, RC48 and CM alone exhibited modest tumor growth inhibition, and their TGI values were 46.04% and 54.26%, respectively. However, the combination treatment of RC48 and CM significantly inhibited tumor growth, resulting in a TGI of 75.55% (Fig. 7A). Similarly, in the OVCAR-3 CDX model, RC48 alone exhibited minimal inhibitory effects with a TGI of 20.45%, while CM showed slight antitumor activity with a TGI of 30.95%. In contrast, the combination treatment resulted in a remarkable synergistic tumor inhibition rate of 65.96% (Fig. 7B).

Furthermore, at the end of the experiment, all CDX xenograft tumors were collected and subjected to immunohistochemistry staining for Ki-67 expression and apoptosis analysis using the TUNEL assay. The immunohistochemistry staining demonstrated that the combination of RC48 and CM led to a significant decrease in Ki-67 expression compared to all other single-agent groups, indicating enhanced antiproliferative activity of the combination treatment in both CDX models (Fig. 7C). The quantification of Ki-67 staining intensity further supported the observed decrease in proliferative activity in the combination treatment group compared to the single-agent groups (Fig. 7D). These findings suggest that the combination of RC48 and CM exerts a synergistic effect in inhibiting tumor cell proliferation in the CDX models. Immunofluorescence images revealed that the combination of RC48 and CM significantly increased cell apoptosis compared to the VEH, RC48, or CM alone (Fig. 7E, F). These findings further support the significant inhibitory effect of the combination therapy on OC tumor growth in vivo.

### RC48 synergized remarkably with CM to improve the anti-tumor activity in PDX models

**Fig. 8:**
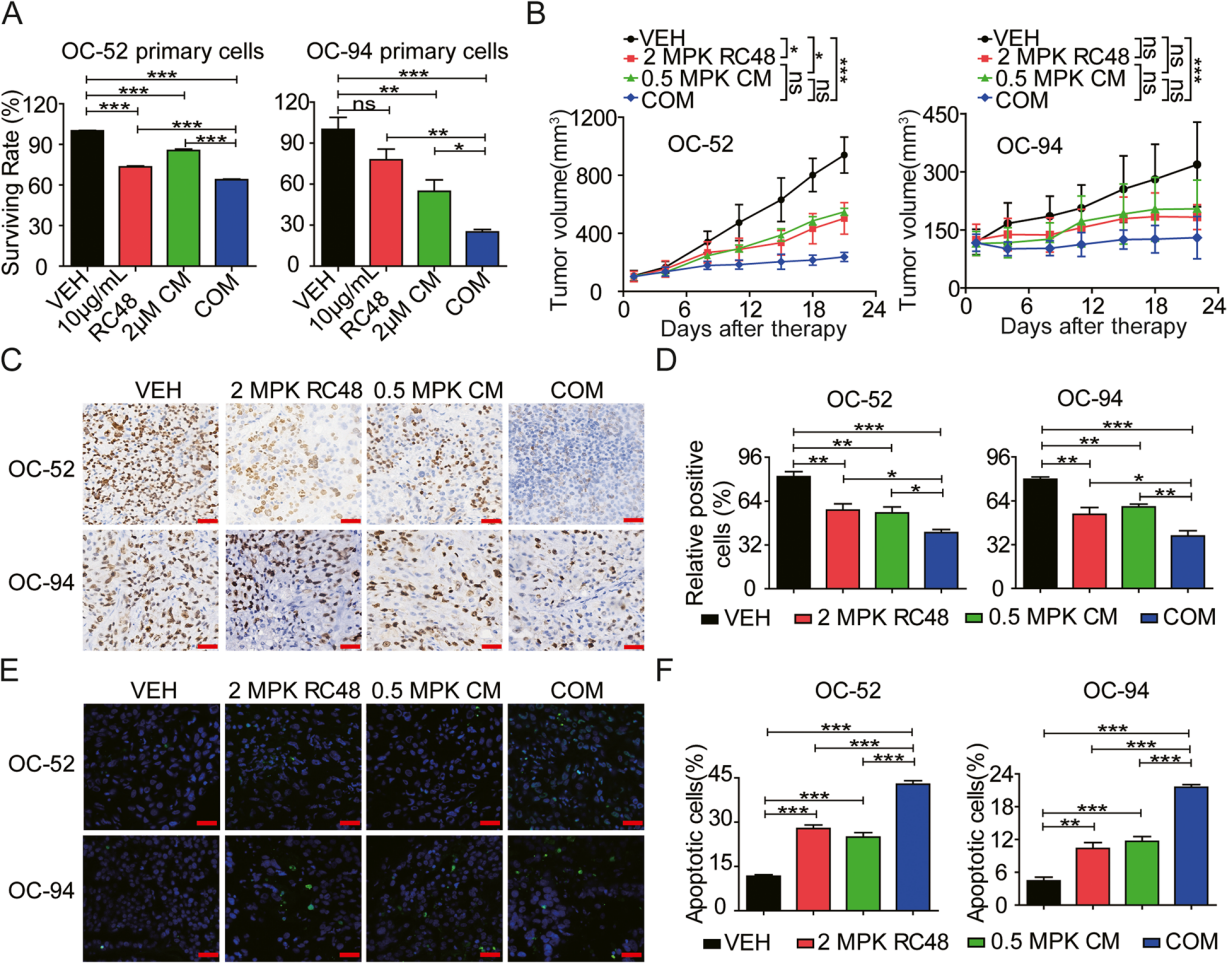
RC48 and CM synergistically inhibit tumor growth in OC PDX models. **A** OC-52 and OC-94 PDX-derived cells were isolated in vitro, and treated with VEH, 10 μg/mL RC48, 2 µM CM or their COM for 5 days. Cell viability was assessed using Cell Titer-Glo cytotoxicity assays. **B** In both PDX models, mice were intravenously administered with VEH, 2.0 MPK RC48 (once a week for 3 times), 0.5 MPK CM (once every day), and their COM. Tumor growth curves were evaluated in OC-52 and OC-94 PDX models. **C**, **D** Representative IHC image of Ki‐67 staining (proliferation index) in OC-52 (upper panel) and OC-94 (lower panel) xenograft tumors. Images were captured with TissueFAXS Plus at 400 × magnification. Scale bars = 20 µm. **E**, **F** The quantification of apoptotic cells in OC-52 (upper panel) and OC-94 (lower panel) xenograft tumors was determined by TUNEL assay. Apoptotic cells are represented by green fluorescent-stained cell nuclei. Images were captured using LSCM at 400 × magnification. Scale bars = 20 µm. The data shown represent the mean ± SEM from three independent experiments performed in triplicate. All treated groups compared to VEH group or COM group compared to single agent group. Statistically significant values are denoted as *p < 0.05, **p < 0.01, and ***p < 0.001

To further investigate the synergistic antitumor effects of the combination treatment of RC48 and CM in vivo, OC-52 and OC-94 PDX models were selected for efficacy evaluation. Cell viability assays demonstrated that the combination of RC48 and CM more effectively inhibited tumor cell growth in both OC-52 and OC-94 PDX-derived primary cells, compared to RC48 or CM alone. Furthermore, the combination treatment showed a stronger inhibitory effect in OC-94 cells than in OC-52 cells (Fig. 8A). In the OC-52 PDX model, mice treated with 2.0 mg/kg RC48 or 0.5 mg/kg CM exhibited a moderate decrease in tumor volume, resulting in tumor growth inhibition (TGI) of 46.47% and 41.59%, respectively, compared to the VEH group. Importantly, the combination of RC48 and CM significantly suppressed tumor growth compared to the monotherapy groups, resulting in a TGI of 74.67% (Fig. 8B).

Similarly, in the OC-94 PDX model, mice treated with 2.0 mg/kg RC48 or 0.5 mg/kg CM also displayed a moderate decrease in tumor volume, with TGIs of 42.56% and 35.86%, respectively, compared to the VEH group. Notably, the combination of 2.0 mg/kg RC48 and 0.5 mg/kg CM exhibited significantly better antitumor activity compared to the monotherapy groups, resulting in a TGI of 59.24% (Fig. 8B). Furthermore, we collected all PDX xenograft tumors and conducted analyses using Western blot, immunohistochemistry, and TUNEL assay. Immunohistochemistry staining for Ki-67, a marker of proliferation, showed that all treatment groups significantly inhibited Ki-67-positive cells compared to the VEH group. Importantly, the combination of RC48 and CM exhibited a more significant inhibitory effect on tumor cell proliferation compared to single agent groups (Fig. 8C, D). Similarly, the TUNEL assay demonstrated significantly higher numbers of apoptosis-positive cells in the groups treated with RC48 or CM alone, as well as the combination therapy group, compared to the vehicle group. Notably, the combination treatment showed superior apoptotic effects when compared to the VEH, RC48, or CM alone (Fig. 8E, F).

In addition, the combination treatment was well-tolerated in both CDX and PDX models, as there were no significant changes in body weight observed throughout the experimental process. H&E staining of essential organs in the CDX and PDX model mice indicated no obvious toxic side effects on the heart, liver, spleen, lung, and kidney compared to the VEH group. These results suggested that the combination therapy had negligible toxicity in vivo (Supplementary Figs. 10 and 11).

Overall, these exciting findings show that combination of RC48 and CM synergistically enhances tumoricidal activity in vivo, providing a strong rationale for further clinical development of the RC48 and CM combination for treating OC.

## Discussion

Ovarian cancer (OC) is a highly heterogeneous disease, and the identification of specific molecular targets has become crucial for developing effective therapeutic strategies. In this study, we focused on the therapeutic efficacy of RC48, an antibody-drug conjugate (ADC) targeting HER2, alone or in combination with CM, an oral VEGFR inhibitor, in advanced OC. Our findings highlight the significance of HER2 expression in OC and demonstrate the synergistic antitumor effects of combining RC48 and CM.

HER2 is commonly observed in patients with the serous subtype and late-stage, highly differentiated OC [61]. Lee et al. reported that HER2 expression is low in normal ovarian epithelial cells but highly expressed in epithelial OC, including 45.5% of mucinous OC, 41.7% of clear cell OC, and 17.5% of serous OC [62]. Studies have also shown that the expression of HER2 in OC is associated with tumor stage, recurrence rate, survival period, and sensitivity to platinum-based chemotherapy [63, 64]. Consistent with previous reports, we confirmed the overexpression of HER2 in human OC cell lines, PDXs, and patient specimens. This observation supports the relevance of HER2 as a therapeutic target in OC.

Our study systematically investigated the antitumor activity of RC48 in OC cells and PDX models. We found that RC48 significantly inhibited human OC growth in a dose-dependent manner in vitro, outperforming T-DM1 and showing better efficacy than T-DXd. Trastuzumab, another anti-HER2 agent, did not show cytotoxicity in these HER2-positive OC cells. Notably, RC48 alone demonstrated excellent tumoricidal activity in OC PDX models. These findings suggest that RC48 is more effective than other anti-HER2 agents in these OC cells.

In addition to investigating the effects of RC48, we screened 22 VEGFR inhibitors in various stages of research and development. Among them, CM exhibited much stronger antiproliferative activity compared to other VEGFR inhibitors. Our data suggested that combining CM with other agents could potentially improve its efficacy, reduce lesion size, and prolong overall survival. Although CM monotherapy has only shown modest clinical benefits in advanced OC and melanoma in phase II/III studies [43, 65, 66], our findings provide a rationale for exploring combination strategies to enhance its therapeutic potential.

ADC monotherapy has shown promising anti-tumor effects in various hematological and solid cancers. However, resistance mechanisms often limit the duration of objective responses or clinical benefits [67, 68]. While ADC combination therapy has been extensively explored with chemotherapy and immune checkpoint inhibitors, there are limited clinical trials investigating the combination of ADCs with targeted therapy or anti-angiogenic inhibitors. In our previous study, we reported the superior antitumor activity of the novel anti-DR5 ADC, Oba01, in combination with gemcitabine in DR5-positive CDX and PDX models, without inducing toxic effects or tolerance [69]. These findings highlight the potential of ADC combination therapy and its importance in improving treatment efficacy in OC. However, there are currently limited clinical trials exploring the combination of ADCs with targeted therapy, immunotherapy, or anti-angiogenic inhibitors. For instance, ongoing clinical trials are evaluating the combination of T-DM1 with alpelisib, tucatinib, or neratinib in patients with metastatic breast cancer [70–72].

In this study, we investigated the combined anti-tumor activity of RC48 and CM in OC. Importantly, we found that the combination of RC48 and CM exhibited highly synergistic antitumor activity against OC cells in vitro. The therapeutic potential of this combination treatment was further supported by in vivo studies, which demonstrated significantly enhanced synergistic effects in RC48 combined with CM-treated CDX and PDX models compared to either single agent. Notably, this combination treatment did not show adverse effects.

To understand the underlying mechanisms of the synergistic effects of RC48 and CM combination treatment in OC cells, we evaluated their impact on apoptosis and cell motility. The combination of RC48 and CM significantly induced apoptosis in A2780 and OVCAR-3 cells, as evidenced by the regulation of apoptotic proteins such as c-Myc, BAX, and MCl-1. Moreover, the combination treatment exhibited significant inhibitory effects on migration and invasion in A2780 and OVCAR-3 cells, accompanied by changes in the expression of epithelial-to-mesenchymal transition (EMT) markers including ZEB1, N-cadherin, and Snail1.

Furthermore, RNA-seq data analysis revealed that RC48 and CM combination treatment affected key cancer-related pathways, including the mTOR signaling pathway, MAPK signaling pathway, FOXO signaling pathway, cell cycle, and p53 signaling pathway. These pathways collectively contributed to the observed in vitro anti-tumor effect. The combination treatment showed a greater contribution of RC48 in A2780 cells and CM in OVCAR-3 cells. Additionally, key genes involved in the FOXO pathway, cell cycle, p53 pathway, AMPK pathway, apoptosis, and lysosome were significantly altered in the combination treatment group. Consistent with the RNA-seq analysis, the protein expression of p-FOXO3A, and CDC25C was downregulated by the combination treatment of RC48 and CM. Similarly, the levels of p-HER2, p-AKT, and p-mTOR were also downregulated in OC cells. These findings provide mechanistic insights into the synergistic effects of the combination treatment.

Moreover, gene set enrichment analysis identified important gene sets regulated by the combination therapy, including genes involved in ferroptosis, lysosomes, cellular senescence, GAP junction, hippo signaling pathway, and TGF-β signaling pathway. Interestingly, the overexpression of certain genes (SMAD3, ZFYVE16, BMP6, LEFTY1, and DVL2) and the low expression of others (CDKN2B and WNT11) were associated with favorable prognosis in OC patients. These genes have been implicated in tumor cell growth, proliferation, invasion, and metastasis in various cancers [73–78].

Looking ahead, future research should focus on further elucidating the mechanisms underlying the synergistic effects of RC48 and CM combination treatment. Additionally, clinical trials are warranted to evaluate the efficacy and safety of this combination therapy in OC patients. The identification and validation of predictive biomarkers for patient stratification and response assessment should be an integral part of future studies. Moreover, exploring novel combination strategies involving ADCs and targeted therapies or anti-angiogenic inhibitors could lead to improved treatment outcomes. It is important to note that the dual targeting of HER2 and VEGFR using combination therapy has the potential to be extended beyond OC. The aberrant expression of HER2 and VEGFR is observed in several other solid tumors, providing a rationale for further preclinical and clinical investigations in these tumor types. The findings from our study may serve as a foundation for future studies exploring the efficacy of dual-target combination therapy in other HER2- and VEGFR-positive malignancies.

The findings of this study have important clinical implications. The highly synergistic antitumor effects observed with RC48 and CM combination therapy provide a strong rationale for its translation into clinical practice. If validated in clinical trials, this combination therapy could potentially improve treatment outcomes for patients with advanced OC, offering a more effective and personalized therapeutic approach. Furthermore, the identification of gene sets associated with favorable prognosis in OC patients could aid in the development of precision medicine approaches for patient stratification and treatment selection.

## Conclusions

Our study demonstrates the therapeutic effectiveness of combining RC48 and CM in advanced OC. The combination treatment exhibits highly synergistic antitumor activity, induces apoptosis, and inhibits cell motility. The mechanistic analysis reveals the impact of the combination treatment on key cancer-related pathways and identifies important gene sets regulated by the therapy. These findings support the potential of ADC combination therapy as a promising approach for the treatment of OC. Future research and clinical trials should further explore this combination treatment strategy, with a focus on understanding the underlying mechanisms and translating the findings into improved patient care and treatment strategies.

### Supplementary Information

**Supplementary Material 1.**

## Data Availability

Data are available on reasonable request. The data used to support the findings of this study are available from the corresponding author on request.

## Abbreviations

OC: Ovarian cancer
HER2: Human epidermal growth factor receptor-2
VEGFR: Vascular endothelial growth factor receptor
PARP: Poly (ADP-ribose) polymerase
PFS: Progression-free survival
OS: Overall survival
ADC: Antibody drug conjugates
T-DM1: Ado-trastuzumab emtansine
T-DXd: Trastuzumab deruxtecan
RC48: Disitamab vedotin
MMAE: Monomethyl auristatin E
CM: Cediranib Maleate
CTG: Cell TiterGlo
MIR: Maximum-dose inhibitory rate
EMT: Epithelial to mesenchymal transition
RNA-seq: Bulk RNA sequencing
CDX: Cell-derived xenografts
PDX: Patient-derived xenografts
QW: Once a week
TGI: Tumor growth inhibition
TUNEL: Terminal-deoxynucleotidyl transferase mediated nick end labeling
IF: Immunofluorescence
IHC: Immunohistochemistry
PI3K: Phosphatidylinositol 3-kinase
AKT: Protein Kinase B
mTOR: Mammalian Target of Rapamycin
MCL-1: Myeloid Cell Leukemin-1
GAPDH: Glyceraldehyde 3-phosphate dehydrogenase
GEPIA: Gene Expression Profiling Interactive Analysis
DEG: Differentially expressed gene
KEGG: Kyoto Encyclopedia of Genes and Genomes
GSEA: Gene set enrichment analysis

## References

[1] Essel KG, Moore KN (2018). Niraparib for the treatment of ovarian cancer. Expert Rev Anticancer Ther.

[2] Stewart C, Ralyea C, Lockwood S (2019). Ovarian cancer: an integrated review. Semin Oncol Nurs.

[3] Wilson MK, Pujade-Lauraine E, Aoki D, Mirza MR, Lorusso D, Oza AM, du Bois A, Vergote I, Reuss A, Bacon M (2017). Fifth ovarian cancer consensus conference of the Gynecologic Cancer InterGroup: recurrent disease. Ann Oncol.

[4] Kuroki L, Guntupalli SR (2020). Treatment of epithelial ovarian cancer. BMJ.

[5] Arora T, Mullangi S, Lekkala MR. Ovarian cancer. In: StatPearls. Treasure Island: Ineligible companies; 2023. Disclosure: Sanjana Mullangi declares no relevant financial relationships with ineligible companies. Disclosure: Manidhar Reddy Lekkala declares no relevant financial relationships with ineligible companies.

[6] Singh N, Badrun D, Ghatage P (2020). State of the art and up-and-coming angiogenesis inhibitors for ovarian cancer. Expert Opin Pharmacother.

[7] McMullen M, Madariaga A, Lheureux S (2021). New approaches for targeting platinum-resistant ovarian cancer. Semin Cancer Biol.

[8] Pujade-Lauraine E, Fujiwara K, Ledermann JA, Oza AM, Kristeleit R, Ray-Coquard IL, Richardson GE, Sessa C, Yonemori K, Banerjee S (2021). Avelumab alone or in combination with chemotherapy versus chemotherapy alone in platinum-resistant or platinum-refractory ovarian cancer (JAVELIN Ovarian 200): an open-label, three-arm, randomised, phase 3 study. Lancet Oncol.

[9] Tolcher A, Hamilton E, Coleman RL (2023). The evolving landscape of antibody-drug conjugates in gynecologic cancers. Cancer Treat Rev.

[10] Oaknin A, Bosse TJ, Creutzberg CL, Giornelli G, Harter P, Joly F, Lorusso D, Marth C, Makker V, Mirza MR (2022). Endometrial cancer: ESMO Clinical Practice Guideline for diagnosis, treatment and follow-up. Ann Oncol.

[11] Kandalaft LE, Dangaj Laniti D, Coukos G (2022). Immunobiology of high-grade serous ovarian cancer: lessons for clinical translation. Nat Rev Cancer.

[12] Erickson BK, Zeybek B, Santin AD, Fader AN (2020). Targeting human epidermal growth factor receptor 2 (HER2) in gynecologic malignancies. Curr Opin Obstet Gynecol.

[13] Oh DY, Bang YJ (2020). HER2-targeted therapies - a role beyond breast cancer. Nat Rev Clin Oncol.

[14] Hynes NE, Lane HA (2005). ERBB receptors and cancer: the complexity of targeted inhibitors. Nat Rev Cancer.

[15] Swain SM, Shastry M, Hamilton E (2023). Targeting HER2-positive breast cancer: advances and future directions. Nat Rev Drug Discov.

[16] Vergara D, Bellomo C, Zhang X, Vergaro V, Tinelli A, Lorusso V, Rinaldi R, Lvov YM, Leporatti S, Maffia M (2012). Lapatinib/Paclitaxel polyelectrolyte nanocapsules for overcoming multidrug resistance in ovarian cancer. Nanomedicine.

[17] Weroha SJ, Oberg AL, Ziegler KL, Dakhilm SR, Rowland KM, Hartmann LC, Moore DF, Keeney GL, Peethambaram PP, Haluska P (2011). Phase II trial of lapatinib and topotecan (LapTop) in patients with platinum-refractory/resistant ovarian and primary peritoneal carcinoma. Gynecol Oncol.

[18] Minkovsky N, Berezov A (2008). BIBW-2992, a dual receptor tyrosine kinase inhibitor for the treatment of solid tumors. Curr Opin Investig Drugs.

[19] Burstein HJ, Sun Y, Dirix LY, Jiang Z, Paridaens R, Tan AR, Awada A, Ranade A, Jiao S, Schwartz G (2010). Neratinib, an irreversible ErbB receptor tyrosine kinase inhibitor, in patients with advanced ErbB2-positive breast cancer. J Clin Oncol.

[20] McCorkle JR, Gorski JW, Liu J, Riggs MB, McDowell AB, Lin N, Wang C, Ueland FR, Kolesar JM (2021). Lapatinib and poziotinib overcome ABCB1-mediated paclitaxel resistance in ovarian cancer. PLoS One.

[21] Faratian D, Zweemer AJ, Nagumo Y, Sims AH, Muir M, Dodds M, Mullen P, Um I, Kay C, Hasmann M (2011). Trastuzumab and pertuzumab produce changes in morphology and estrogen receptor signaling in ovarian cancer xenografts revealing new treatment strategies. Clin Cancer Res.

[22] Bookman MA, Darcy KM, Clarke-Pearson D, Boothby RA, Horowitz IR (2003). Evaluation of monoclonal humanized anti-HER2 antibody, trastuzumab, in patients with recurrent or refractory ovarian or primary peritoneal carcinoma with overexpression of HER2: a phase II trial of the Gynecologic Oncology Group. J Clin Oncol.

[23] Gordon MS, Matei D, Aghajanian C, Matulonis UA, Brewer M, Fleming GF, Hainsworth JD, Garcia AA, Pegram MD, Schilder RJ (2006). Clinical activity of pertuzumab (rhuMAb 2C4), a HER dimerization inhibitor, in advanced ovarian cancer: potential predictive relationship with tumor HER2 activation status. J Clin Oncol.

[24] Fu Z, Li S, Han S, Shi C, Zhang Y (2022). Antibody drug conjugate: the “biological missile” for targeted cancer therapy. Signal Transduct Target Ther.

[25] Tolcher AW (2022). Antibody drug conjugates: the dos and don’ts in clinical development. Pharmacol Ther.

[26] Montemurro F, Delaloge S, Barrios CH, Wuerstlein R, Anton A, Brain E, Hatschek T, Kelly CM, Pena-Murillo C, Yilmaz M (2020). Trastuzumab emtansine (T-DM1) in patients with HER2-positive metastatic breast cancer and brain metastases: exploratory final analysis of cohort 1 from KAMILLA, a single-arm phase IIIb clinical trial(☆). Ann Oncol.

[27] Shi F, Liu Y, Zhou X, Shen P, Xue R, Zhang M (2022). Disitamab vedotin: a novel antibody-drug conjugates for cancer therapy. Drug Deliv.

[28] Menderes G, Bonazzoli E, Bellone S, Altwerger G, Black JD, Dugan K, Pettinella F, Masserdotti A, Riccio F, Bianchi A (2017). Superior in vitro and in vivo activity of trastuzumab-emtansine (T-DM1) in comparison to trastuzumab, pertuzumab and their combination in epithelial ovarian carcinoma with high HER2/neu expression. Gynecol Oncol.

[29] Jhaveri KL, Wang XV, Makker V, Luoh SW, Mitchell EP, Zwiebel JA, Sharon E, Gray RJ, Li S, McShane LM (2019). Ado-trastuzumab emtansine (T-DM1) in patients with HER2-amplified tumors excluding breast and gastric/gastroesophageal junction (GEJ) adenocarcinomas: results from the NCI-MATCH trial (EAY131) subprotocol Q. Ann Oncol.

[30] Nakada T, Sugihara K, Jikoh T, Abe Y, Agatsuma T (2019). The latest research and development into the antibody-drug conjugate, [fam-] trastuzumab deruxtecan (DS-8201a), for HER2 cancer therapy. Chem Pharm Bull (Tokyo).

[31] Modi S, Park H, Murthy RK, Iwata H, Tamura K, Tsurutani J, Moreno-Aspitia A, Doi T, Sagara Y, Redfern C (2020). Antitumor activity and safety of trastuzumab deruxtecan in patients with HER2-low-expressing advanced breast cancer: results from a phase Ib study. J Clin Oncol.

[32] Peng Z, Liu T, Wei J, Wang A, He Y, Yang L, Zhang X, Fan N, Luo S, Li Z (2021). Efficacy and safety of a novel anti-HER2 therapeutic antibody RC48 in patients with HER2-overexpressing, locally advanced or metastatic gastric or gastroesophageal junction cancer: a single-arm phase II study. Cancer Commun (Lond).

[33] Sheng X, Yan X, Wang L, Shi Y, Yao X, Luo H, Shi B, Liu J, He Z, Yu G (2021). Open-label, multicenter, phase II study of RC48-ADC, a HER2-targeting antibody-drug conjugate, in patients with locally advanced or metastatic urothelial carcinoma. Clin Cancer Res.

[34] Xu Y, Wang Y, Gong J, Zhang X, Peng Z, Sheng X, Mao C, Fan Q, Bai Y, Ba Y (2021). Phase I study of the recombinant humanized anti-HER2 monoclonal antibody-MMAE conjugate RC48-ADC in patients with HER2-positive advanced solid tumors. Gastric Cancer.

[35] Jiang J, Dong L, Wang L, Wang L, Zhang J, Chen F, Zhang X, Huang M, Li S, Ma W (2016). HER2-targeted antibody drug conjugates for ovarian cancer therapy. Eur J Pharm Sci.

[36] Mariotti V, Fiorotto R, Cadamuro M, Fabris L, Strazzabosco M (2021). New insights on the role of vascular endothelial growth factor in biliary pathophysiology. JHEP Rep.

[37] Goel HL, Mercurio AM (2013). VEGF targets the tumour cell. Nat Rev Cancer.

[38] Olsson AK, Dimberg A, Kreuger J, Claesson-Welsh L (2006). VEGF receptor signalling - in control of vascular function. Nat Rev Mol Cell Biol.

[39] Simons M, Gordon E, Claesson-Welsh L (2016). Mechanisms and regulation of endothelial VEGF receptor signalling. Nat Rev Mol Cell Biol.

[40] Ivy SP, Wick JY, Kaufman BM (2009). An overview of small-molecule inhibitors of VEGFR signaling. Nat Rev Clin Oncol.

[41] Monk BJ, Dalton H, Farley JH, Chase DM, Benjamin I (2013). Antiangiogenic agents as a maintenance strategy for advanced epithelial ovarian cancer. Crit Rev Oncol Hematol.

[42] Orbegoso C, Marquina G, George A, Banerjee S (2017). The role of Cediranib in ovarian cancer. Expert Opin Pharmacother.

[43] Matulonis UA, Berlin S, Ivy P, Tyburski K, Krasner C, Zarwan C, Berkenblit A, Campos S, Horowitz N, Cannistra SA (2009). Cediranib, an oral inhibitor of vascular endothelial growth factor receptor kinases, is an active drug in recurrent epithelial ovarian, fallopian tube, and peritoneal cancer. J Clin Oncol.

[44] Hirte H, Lheureux S, Fleming GF, Sugimoto A, Morgan R, Biagi J, Wang L, McGill S, Ivy SP, Oza AM (2015). A phase 2 study of cediranib in recurrent or persistent ovarian, peritoneal or fallopian tube cancer: a trial of the Princess Margaret, Chicago and California Phase II Consortia. Gynecol Oncol.

[45] Mahner S, Woelber L, Mueller V, Witzel I, Prieske K, Grimm D, Keller VAG, Trillsch F (2015). Beyond bevacizumab: an outlook to new anti-angiogenics for the treatment of ovarian cancer. Front Oncol.

[46] Ledermann JA, Embleton-Thirsk AC, Perren TJ, Jayson GC, Rustin GJS, Kaye SB, Hirte H, Oza A, Vaughan M, Friedlander M (2021). Cediranib in addition to chemotherapy for women with relapsed platinum-sensitive ovarian cancer (ICON6): overall survival results of a phase III randomised trial. ESMO Open.

[47] Liu JF, Barry WT, Birrer M, Lee JM, Buckanovich RJ, Fleming GF, Rimel B, Buss MK, Nattam S, Hurteau J (2014). Combination cediranib and olaparib versus olaparib alone for women with recurrent platinum-sensitive ovarian cancer: a randomised phase 2 study. Lancet Oncol.

[48] Liu JF, Barry WT, Birrer M, Lee JM, Buckanovich RJ, Fleming GF, Rimel BJ, Buss MK, Nattam SR, Hurteau J (2019). Overall survival and updated progression-free survival outcomes in a randomized phase II study of combination cediranib and olaparib versus olaparib in relapsed platinum-sensitive ovarian cancer. Ann Oncol.

[49] Alvarez Secord A, O’Malley DM, Sood AK, Westin SN, Liu JF (2021). Rationale for combination PARP inhibitor and antiangiogenic treatment in advanced epithelial ovarian cancer: a review. Gynecol Oncol.

[50] Zhang J, Yang PL, Gray NS (2009). Targeting cancer with small molecule kinase inhibitors. Nat Rev Cancer.

[51] Gross S, Rahal R, Stransky N, Lengauer C, Hoeflich KP (2015). Targeting cancer with kinase inhibitors. J Clin Invest.

[52] Holohan C, Van Schaeybroeck S, Longley DB, Johnston PG (2013). Cancer drug resistance: an evolving paradigm. Nat Rev Cancer.

[53] Kodack DP, Chung E, Yamashita H, Incio J, Duyverman AM, Song Y, Farrar CT, Huang Y, Ager E, Kamoun W (2012). Combined targeting of HER2 and VEGFR2 for effective treatment of HER2-amplified breast cancer brain metastases. Proc Natl Acad Sci U S A.

[54] Martin M, Makhson A, Gligorov J, Lichinitser M, Lluch A, Semiglazov V, Scotto N, Mitchell L, Tjulandin S (2012). Phase II study of bevacizumab in combination with trastuzumab and capecitabine as first-line treatment for HER-2-positive locally recurrent or metastatic breast cancer. Oncologist.

[55] Tai W, Mahato R, Cheng K (2010). The role of HER2 in cancer therapy and targeted drug delivery. J Control Release.

[56] Hu X, Li D, Fu Y, Zheng J, Feng Z, Cai J, Wang P (2022). Advances in the application of radionuclide-labeled HER2 affibody for the diagnosis and treatment of ovarian cancer. Front Oncol.

[57] Hidalgo M, Amant F, Biankin AV, Budinska E, Byrne AT, Caldas C, Clarke RB, de Jong S, Jonkers J, Maelandsmo GM (2014). Patient-derived xenograft models: an emerging platform for translational cancer research. Cancer Discov.

[58] Risbridger GP, Lawrence MG, Taylor RA (2020). PDX: moving beyond drug screening to versatile models for research discovery. J Endocr Soc.

[59] Lamouille S, Xu J, Derynck R (2014). Molecular mechanisms of epithelial-mesenchymal transition. Nat Rev Mol Cell Biol.

[60] Kalluri R, Weinberg RA (2009). The basics of epithelial-mesenchymal transition. J Clin Invest.

[61] Reibenwein J, Krainer M (2008). Targeting signaling pathways in ovarian cancer. Expert Opin Ther Targets.

[62] Lee ES, Lee Y, Suh D, Kang J, Kim I (2010). Detection of HER-2 and EGFR gene amplification using chromogenic in-situ hybridization technique in ovarian tumors. Appl Immunohistochem Mol Morphol.

[63] Camilleri-Broet S, Hardy-Bessard AC, Le Tourneau A, Paraiso D, Levrel O, Leduc B, Bain S, Orfeuvre H, Audouin J, Pujade-Lauraine E, group G (2004). HER-2 overexpression is an independent marker of poor prognosis of advanced primary ovarian carcinoma: a multicenter study of the GINECO group. Ann Oncol.

[64] Serrano-Olvera A, Duenas-Gonzalez A, Gallardo-Rincon D, Candelaria M, De la Garza-Salazar J (2006). Prognostic, predictive and therapeutic implications of HER2 in invasive epithelial ovarian cancer. Cancer Treat Rev.

[65] McWhirter E, Quirt I, Gajewski T, Pond G, Wang L, Hui J, Oza A (2016). A phase II study of cediranib, an oral VEGF inhibitor, in previously untreated patients with metastatic or recurrent malignant melanoma. Invest New Drugs.

[66] Cohen JW, Widemann BC, Derdak J, Dombi E, Goodwin A, Dompierre J, Onukwubiri U, Steinberg SM, O’Sullivan Coyne G, Kummar S (2019). Cediranib phase-II study in children with metastatic alveolar soft-part sarcoma (ASPS). Pediatr Blood Cancer.

[67] Dumontet C, Reichert JM, Senter PD, Lambert JM, Beck A (2023). Antibody-drug conjugates come of age in oncology. Nat Rev Drug Discov.

[68] Fuentes-Antras J, Genta S, Vijenthira A, Siu LL (2023). Antibody-drug conjugates: in search of partners of choice. Trends Cancer.

[69] Zheng C, Zhou D, Li W, Duan Y, Xu M, Liu J, Cheng J, Xiao Y, Xiao H, Gan T (2023). Therapeutic efficacy of a MMAE-based anti-DR5 drug conjugate Oba01 in preclinical models of pancreatic cancer. Cell Death Dis.

[70] Borges VF, Ferrario C, Aucoin N, Falkson C, Khan Q, Krop I, Welch S, Conlin A, Chaves J, Bedard PL (2018). Tucatinib combined with ado-trastuzumab emtansine in advanced ERBB2/HER2-positive metastatic breast cancer: a phase 1b clinical trial. JAMA Oncol.

[71] Jain S, Shah AN, Santa-Maria CA, Siziopikou K, Rademaker A, Helenowski I, Cristofanilli M, Gradishar WJ (2018). Phase I study of alpelisib (BYL-719) and trastuzumab emtansine (T-DM1) in HER2-positive metastatic breast cancer (MBC) after trastuzumab and taxane therapy. Breast Cancer Res Treat.

[72] Abraham J, Montero AJ, Jankowitz RC, Salkeni MA, Beumer JH, Kiesel BF, Piette F, Adamson LM, Nagy RJ, Lanman RB (2019). Safety and efficacy of T-DM1 plus neratinib in patients with metastatic HER2-positive breast cancer: NSABP foundation trial FB-10. J Clin Oncol.

[73] Lee JH, Lee GT, Woo SH, Ha YS, Kwon SJ, Kim WJ, Kim IY (2013). BMP-6 in renal cell carcinoma promotes tumor proliferation through IL-10-dependent M2 polarization of tumor-associated macrophages. Cancer Res.

[74] Zabala M, Lobo NA, Antony J, Heitink LS, Gulati GS, Lam J, Parashurama N, Sanchez K, Adorno M, Sikandar SS (2020). LEFTY1 is a dual-SMAD inhibitor that promotes mammary progenitor growth and tumorigenesis. Cell Stem Cell.

[75] Deng Z, Cui L, Li P, Ren N, Zhong Z, Tang Z, Wang L, Gong J, Cheng H, Guan Y (2021). Genomic comparison between cerebrospinal fluid and primary tumor revealed the genetic events associated with brain metastasis in lung adenocarcinoma. Cell Death Dis.

[76] Menck K, Heinrichs S, Wlochowitz D, Sitte M, Noeding H, Janshoff A, Treiber H, Ruhwedel T, Schatlo B, von der Brelie C (2021). WNT11/ROR2 signaling is associated with tumor invasion and poor survival in breast cancer. J Exp Clin Cancer Res.

[77] Zheng C, Liu M, Ge Y, Qian Y, Fan H (2022). HBx increases chromatin accessibility and ETV4 expression to regulate dishevelled-2 and promote HCC progression. Cell Death Dis.

[78] Kim JY, Hong N, Park S, Ham SW, Kim EJ, Kim SO, Jang J, Kim Y, Kim JK, Kim SC (2023). Jagged1 intracellular domain/SMAD3 complex transcriptionally regulates TWIST1 to drive glioma invasion. Cell Death Dis.

