# Supplementary material for: Combined inhibition of HER2 and VEGFR synergistically improves therapeutic efficacy via PI3K-AKT pathway in advanced ovarian cancer
Source: J Exp Clin Cancer Res. 2024 Feb 26;43:56. doi: 10.1186/s13046-024-02981-5 (PMC10895844; doi:10.1186/s13046-024-02981-5)
Supplement: Supplementary file 1 — Supplementary Material 1. [file 13046_2024_2981_MOESM1_ESM.docx]

**Supplemental information**


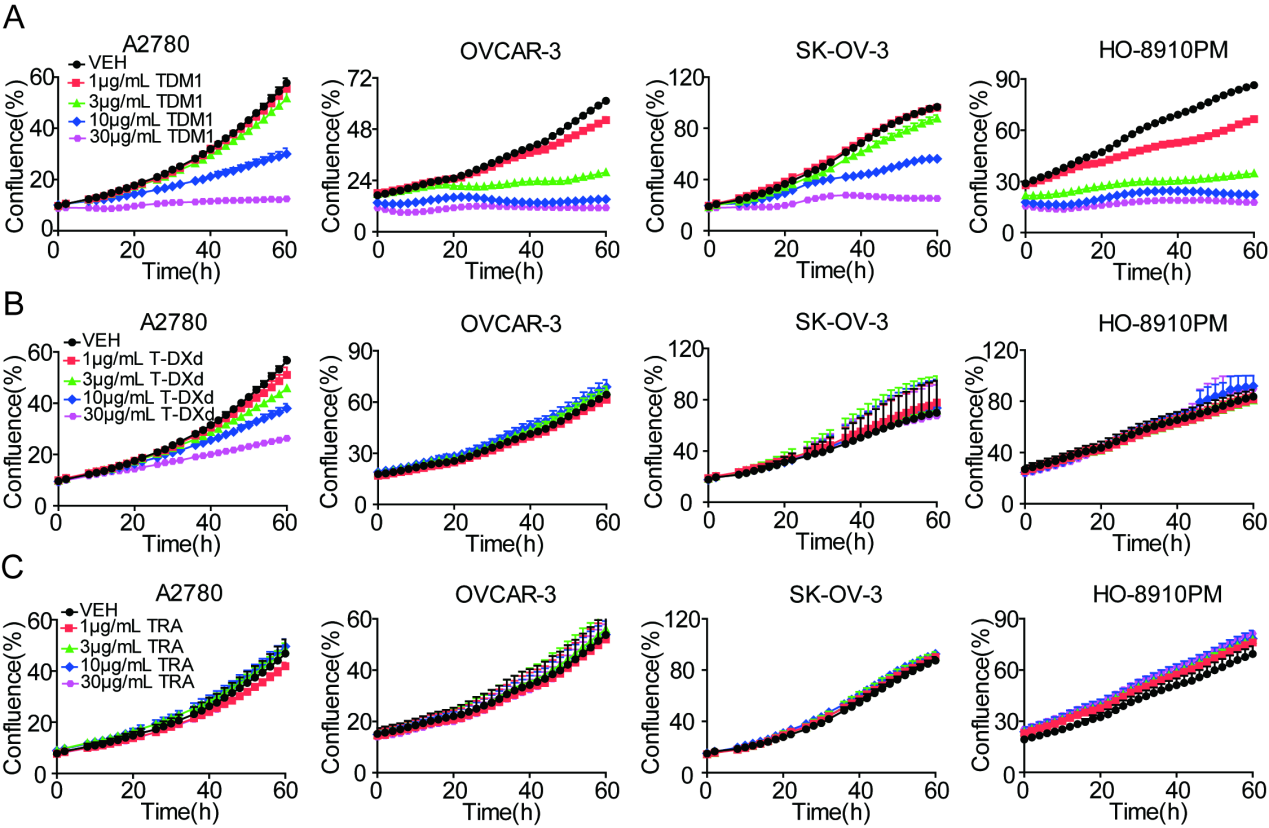


**Supplemental Figure 1**

Anti-tumor effects of T-DM1, T-Dxd and trastuzumab (TRA) on 4 OC cell lines *in vitro*. A2780, OVCAR-3, SK-OV-3, HO-8910PM cells were continuously treated with the indicated drug concentrations for 60 h. Cell proliferation was detected by the IncuCyte^®^ Live cell analysis system and cell confluence curves were automatically generated.


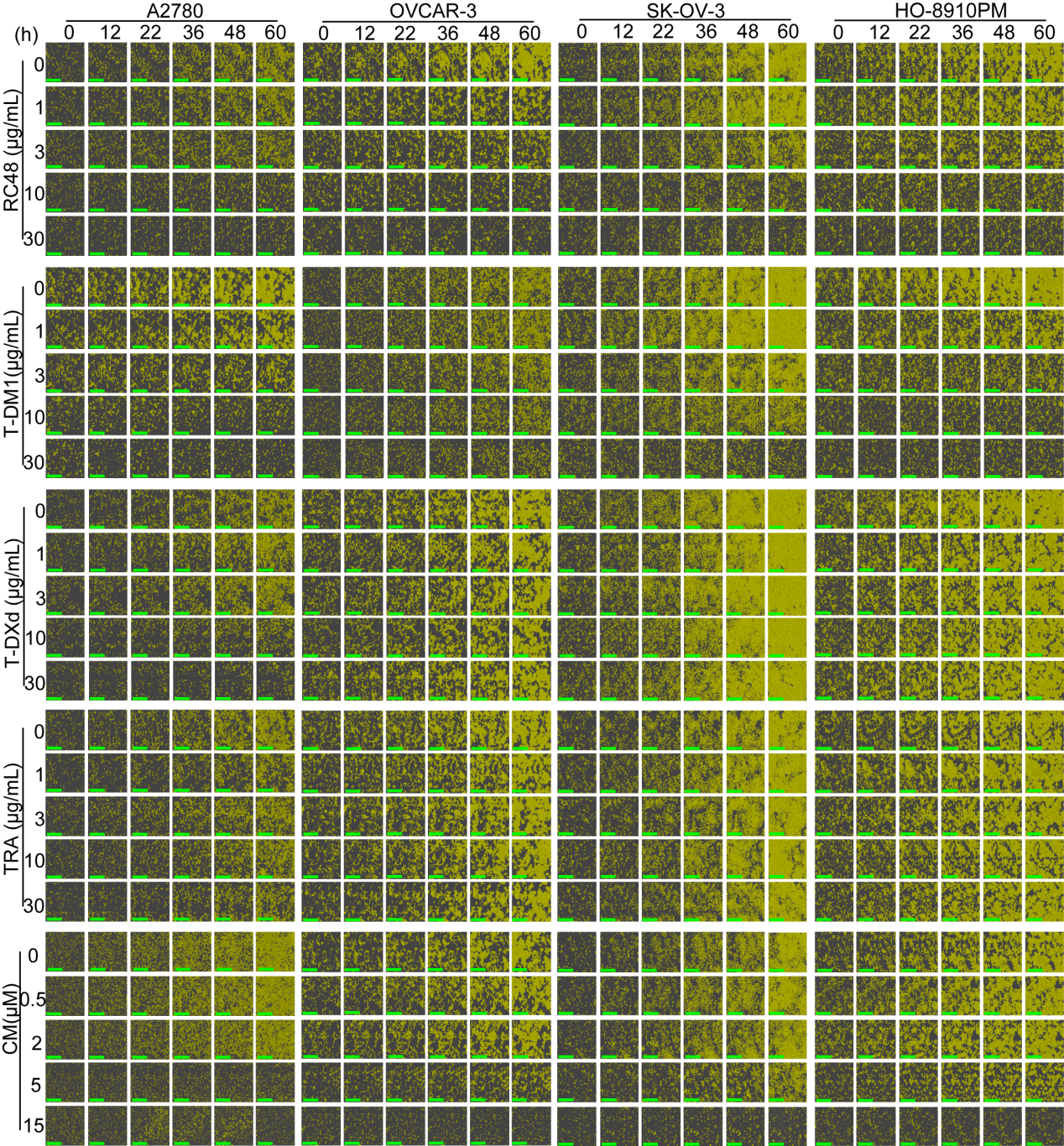


**Supplemental Figure 2**

A2780, OVCAR-3, SK-OV-3, HO-8910PM cells were continuously treated with the RC48, T-DM1, T-Dxd, TRA and CM for 60 h, respectively. IncuCyte^®^ Live cell analysis system acquired cell images at the corresponding time points in real time. Images captured at 100 × magnification, respectively. Scale bars = 400 µm.


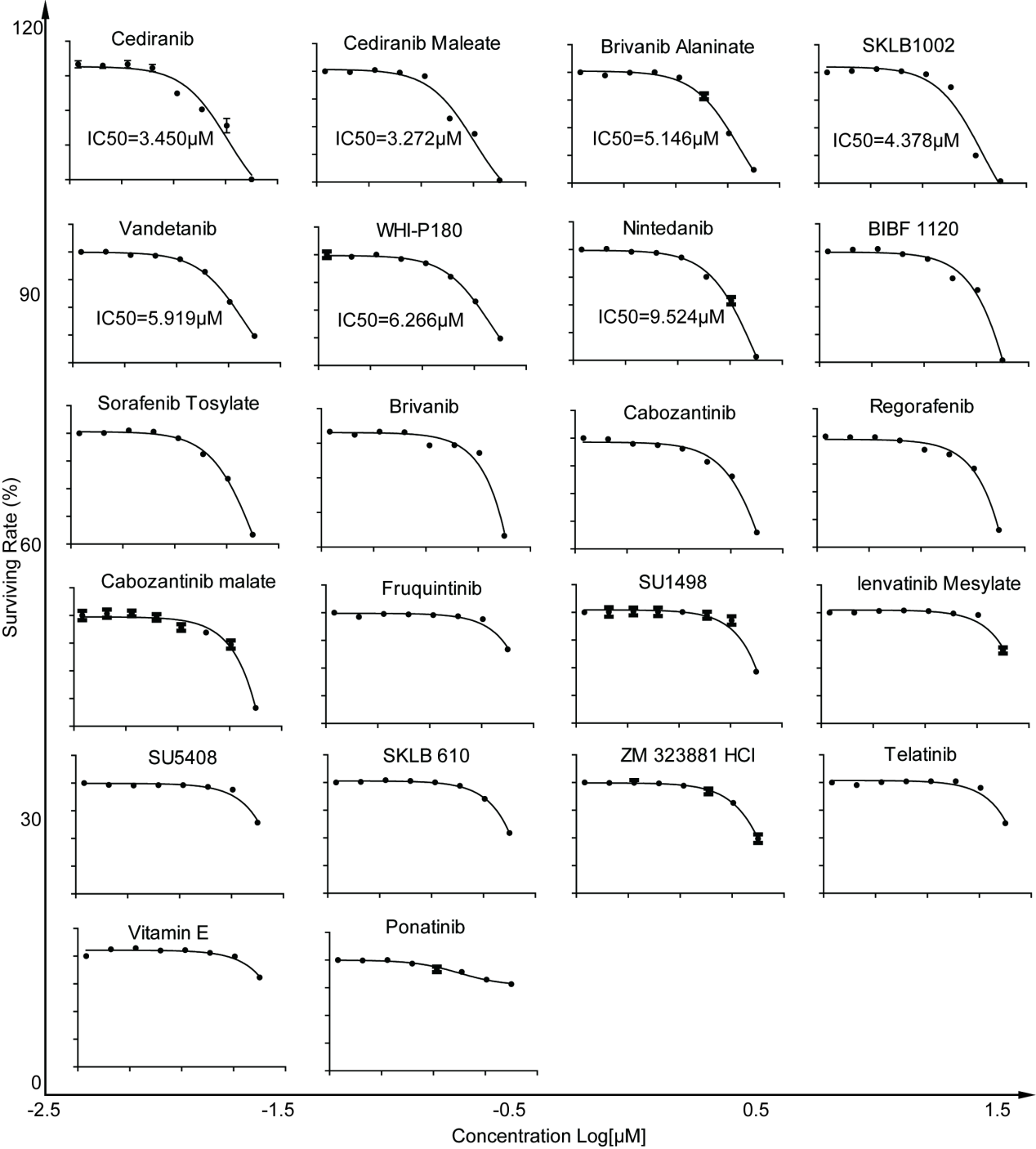


**Supplemental Figure 3**

A2780 cells were treated with 22 VEGFR inhibitors for 72 hours *in vitro*. The cell viability was detected by Cell Titer-Glo^®^ cytotoxicity assay.


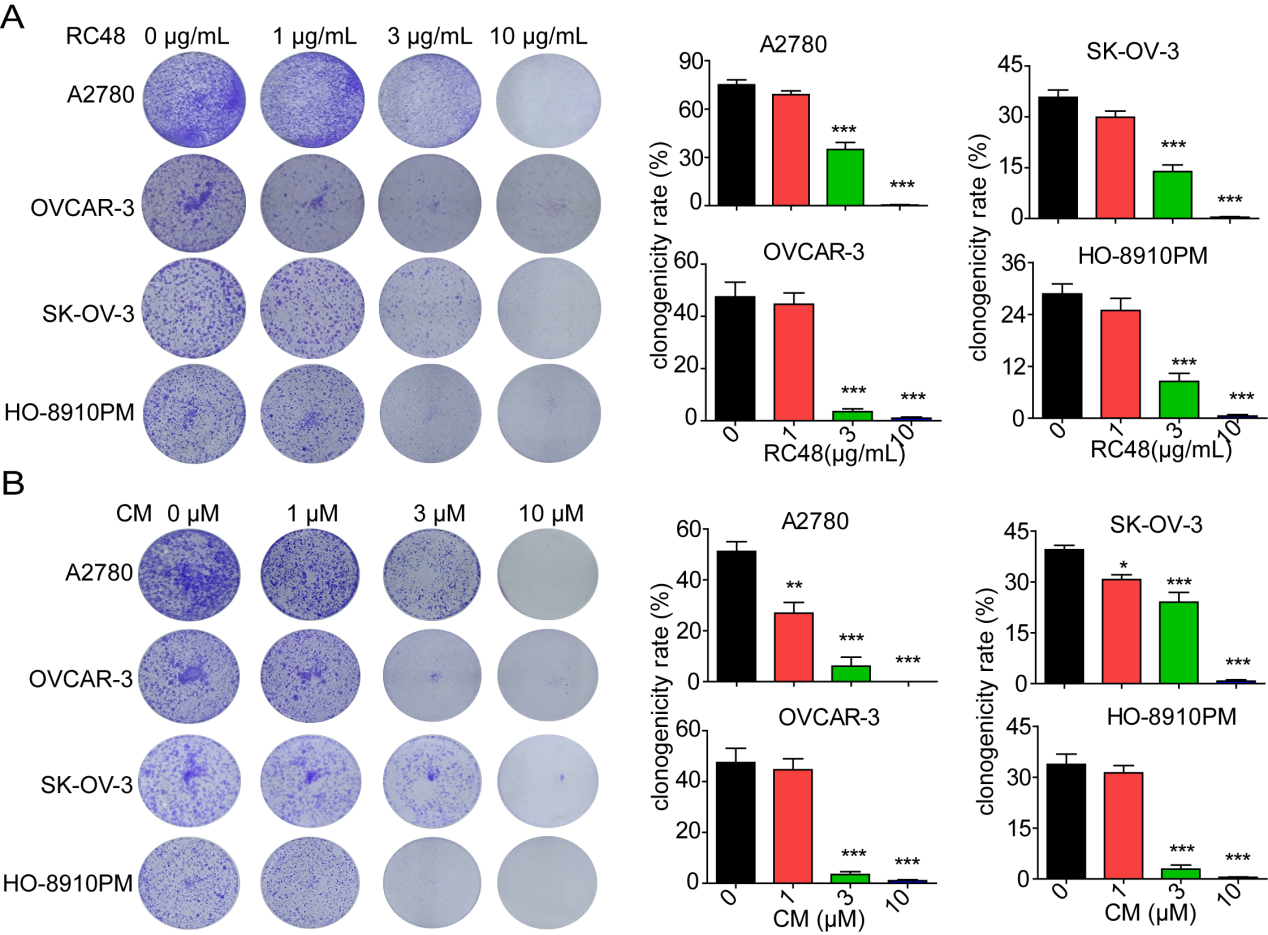


**Supplemental Figure 4**

0, 1, 3, 10 μg/ml RC48 and 0, 1, 3, 10 μM CM was used to treat A2780, OVCAR-3, SKOV-3, HO-8910PM cells at the indicated concentrations for approximately 10 days. The area of colonies stained by crystal violet was used to determine the anti-proliferative effects. Data represent the mean ± SEM of three independent experiments. Statistical significance was assessed by one-way ANOVA with Bonferroni post hoc test (**p* < 0.05; ***p* < 0.01; ****p* < 0.001).


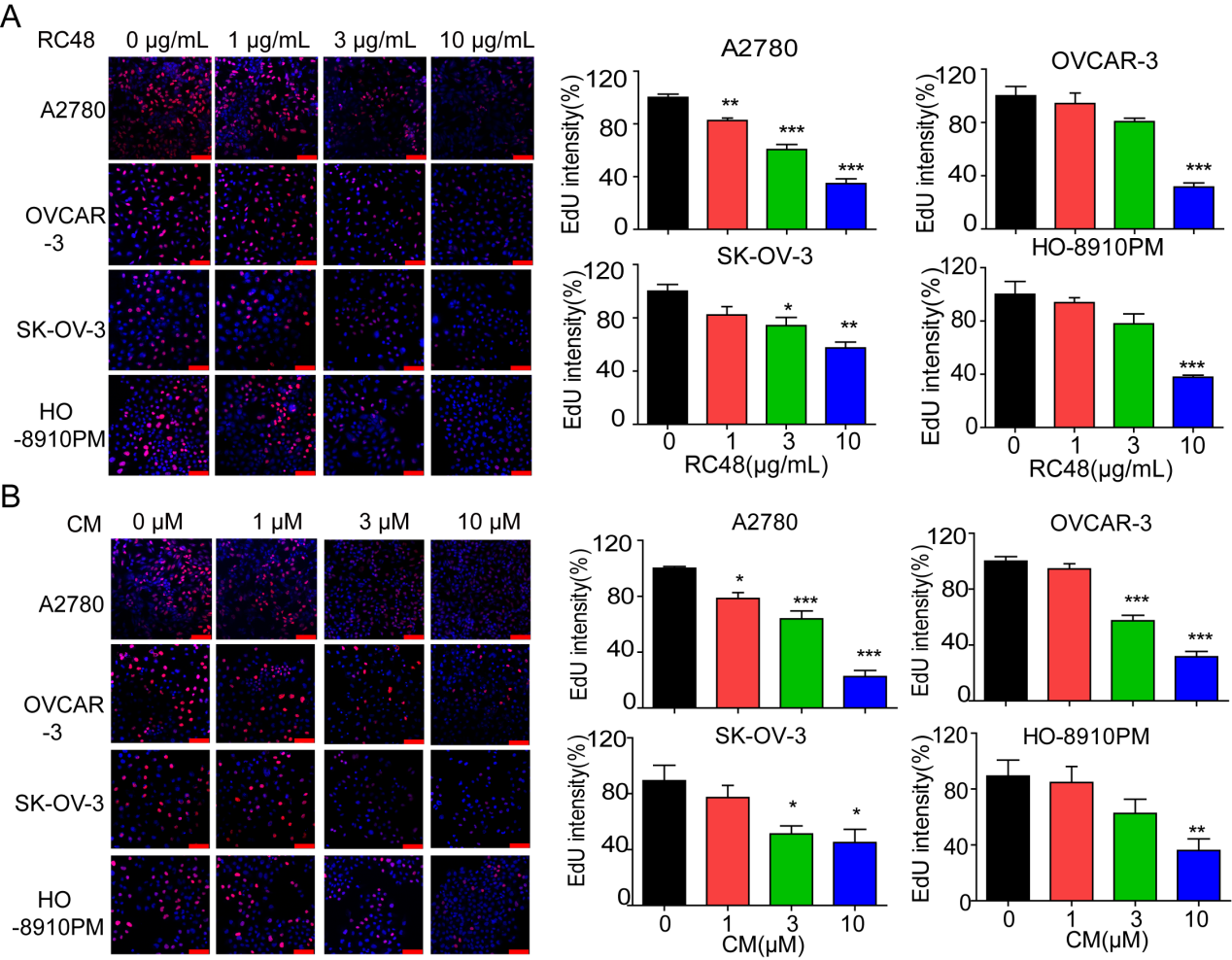


**Supplemental Figure 5**

A2780, OVCAR-3, SKOV-3 and HO-8910PM cells were treated by 0, 1, 3, 10 μg/mL RC48 (A) and 0, 1, 3, 10 μM CM (B) for 24 h, respectively. Then cell proliferation was determined by the EdU assay. Images were captured by the laser scanning confocal microscope. Data are presented as means ± SEM of three independent experiments. Statistical significance was assessed by one-way ANOVA with Bonferroni post hoc test (**p* < 0.05; ***p* < 0.01; ****p* < 0.001). Images captured at 400 × magnification, respectively. Scale bars = 20 µm.


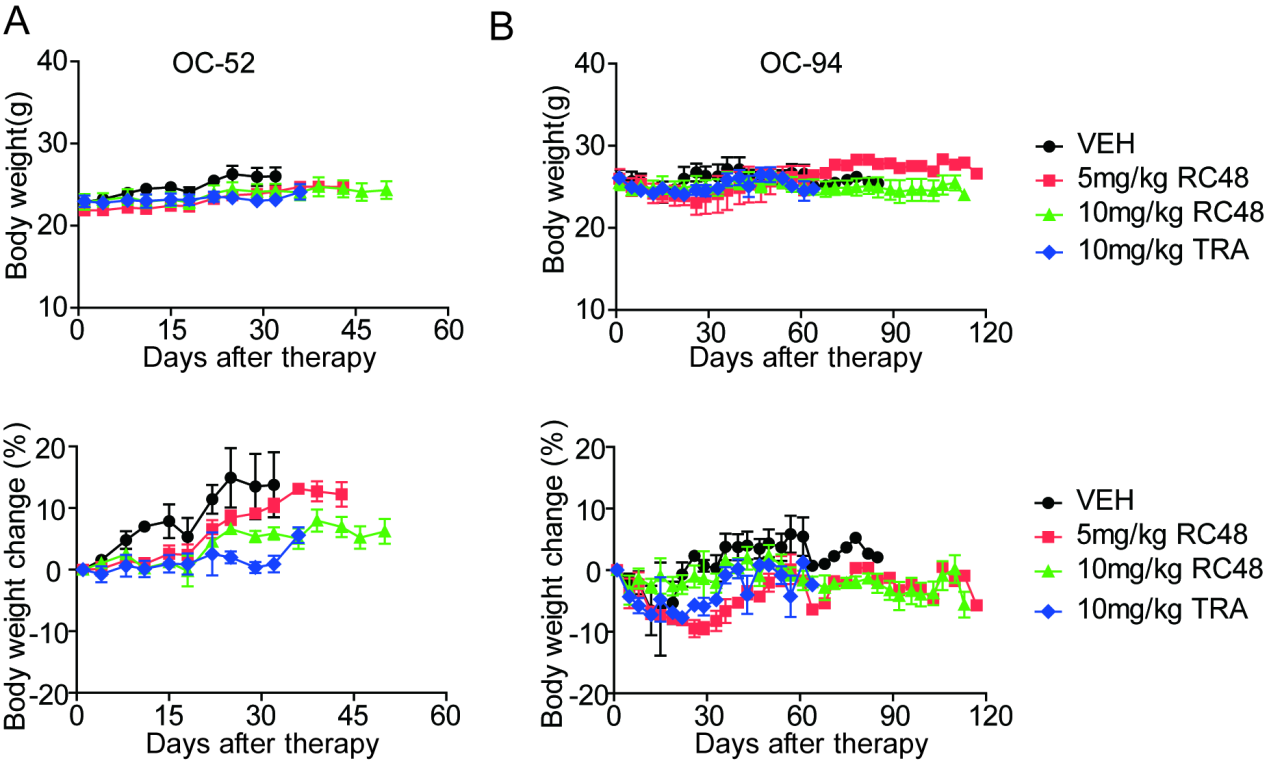


**Supplemental Figure 6**

Mice body weights and the changes of the body weights were assessed twice a week in OvCa52 (A) and OvCa94 (B) PDX models.


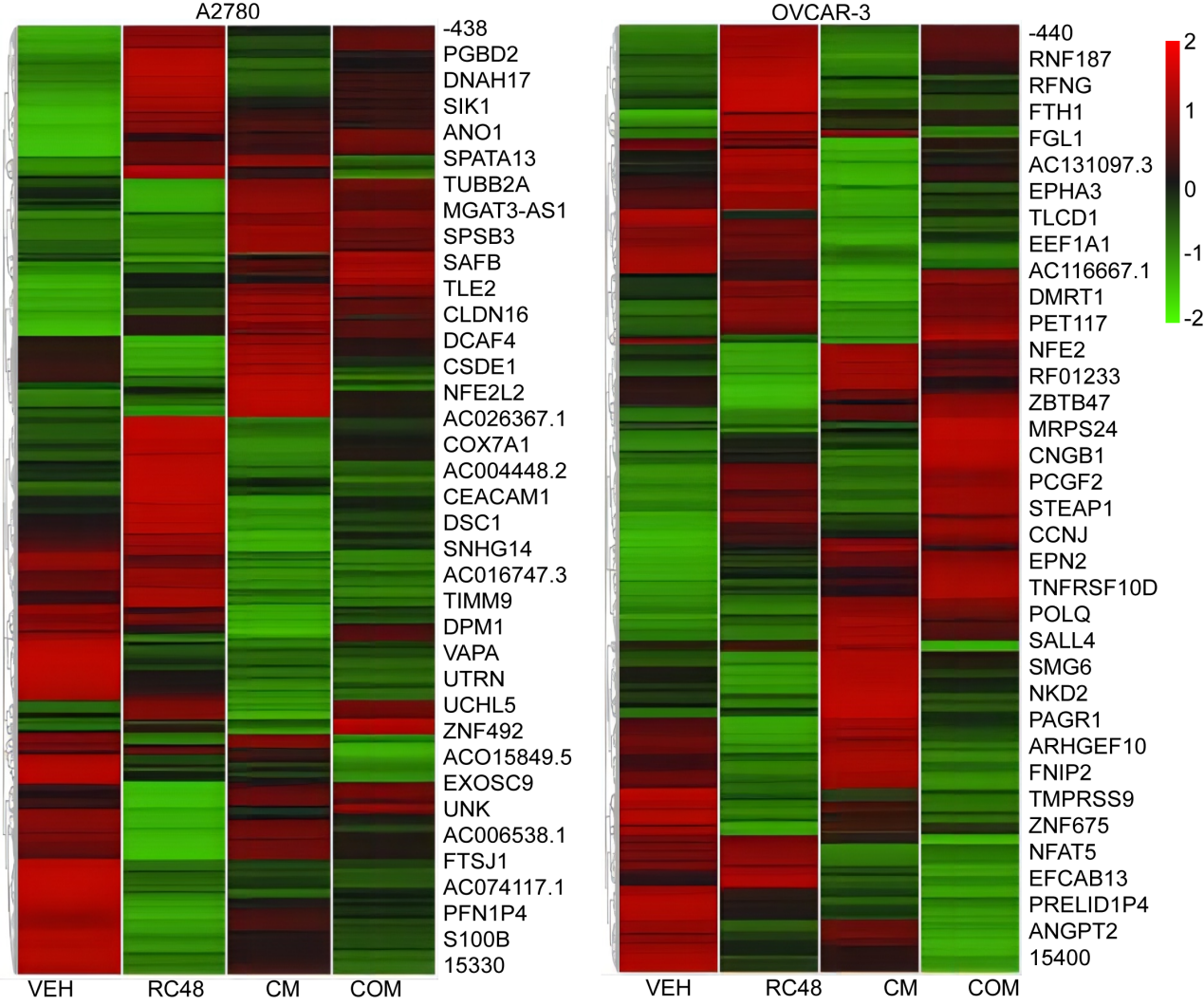


Supplementary Figure 7. Heatmap of a transcriptional profile denoting unbiased clustering in OC cells.

Heatmaps of a transcriptional profile denoting unbiased clustering of A2780 cells and OVCAR-3 cells treated with RC48 and CM singly or in combination (n = 3). Total RNA was analyzed by high-throughput whole transcriptome sequencing (RNA-Seq). Significant differential expression is defined as *P*-value ≤0.05.

**
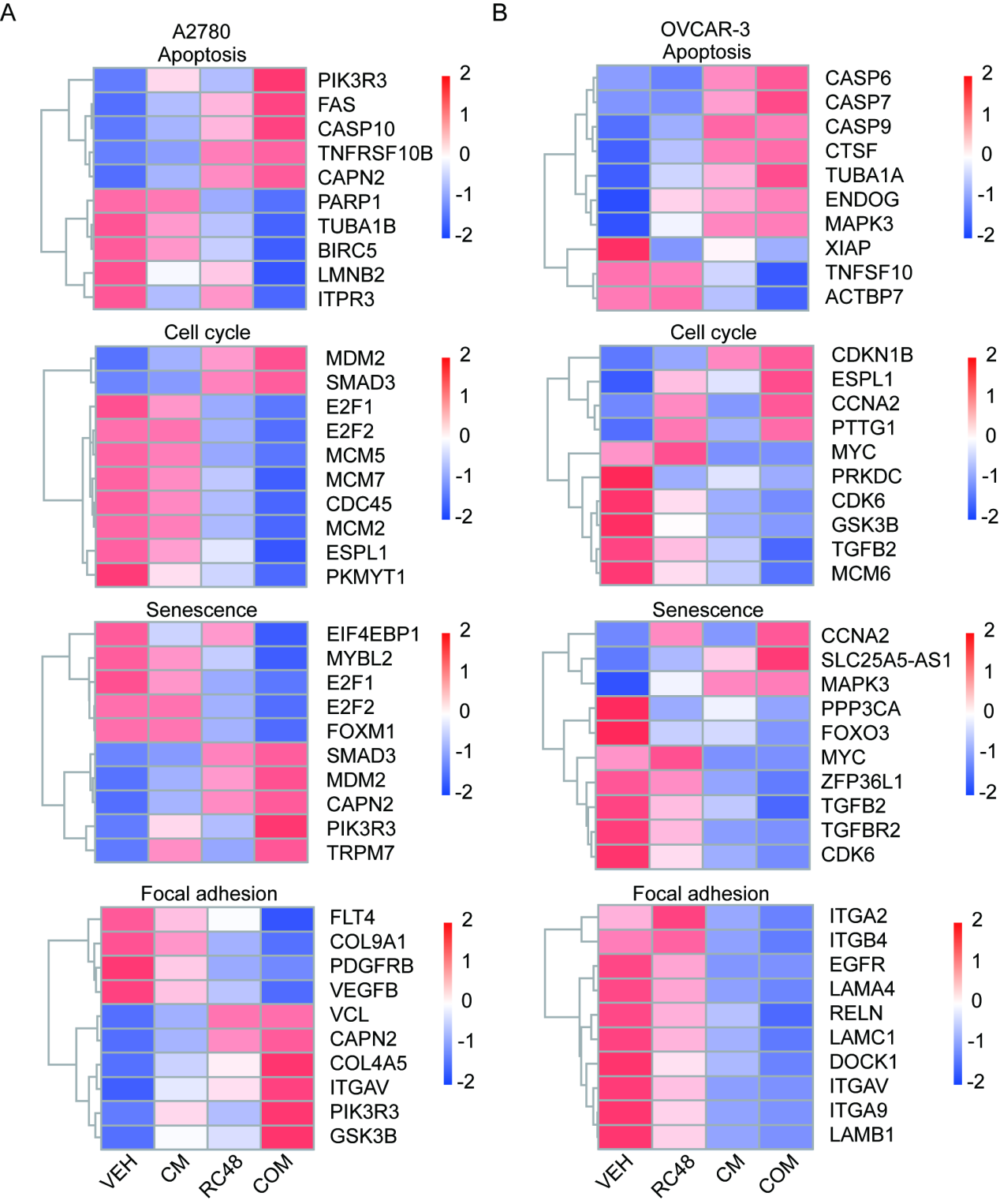
**

Supplementary Figure 8. Combined therapy of RC48 and CM regulated the apoptosis, cell cycle, senescence and focal adhesion DEGs expression in OC cells.

Heatmaps of significantly regulated genes in transcriptomes of A2780 (A) and OVCAR-3 (B) cells correlated with biological processes including apoptosis, the cell cycle, cellular senescence, and focal adhesion (n = 3).


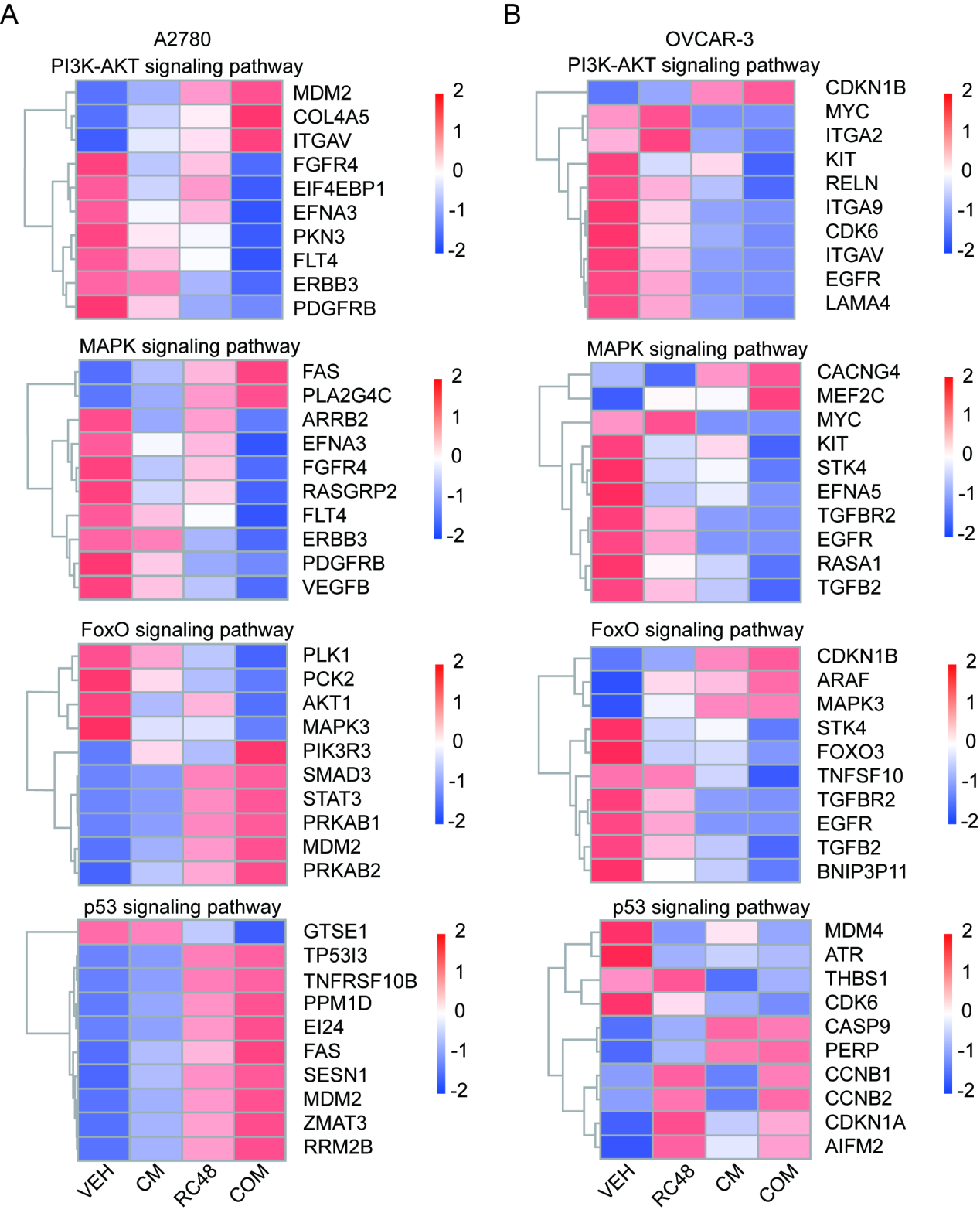


Supplementary Figure 9. Combined therapy of RC48 and CM significantly regulated the related to pathway DEGs expression in OC cells.

Heat maps of significantly regulated genes of transcriptomes of A2780 (A) and OVCAR-3 (B) cells after combinational treatment with RC48 and CM correlated with the PI3K-AKT pathway, MAPK pathway, FoxO pathway and p53 pathway (n = 3).


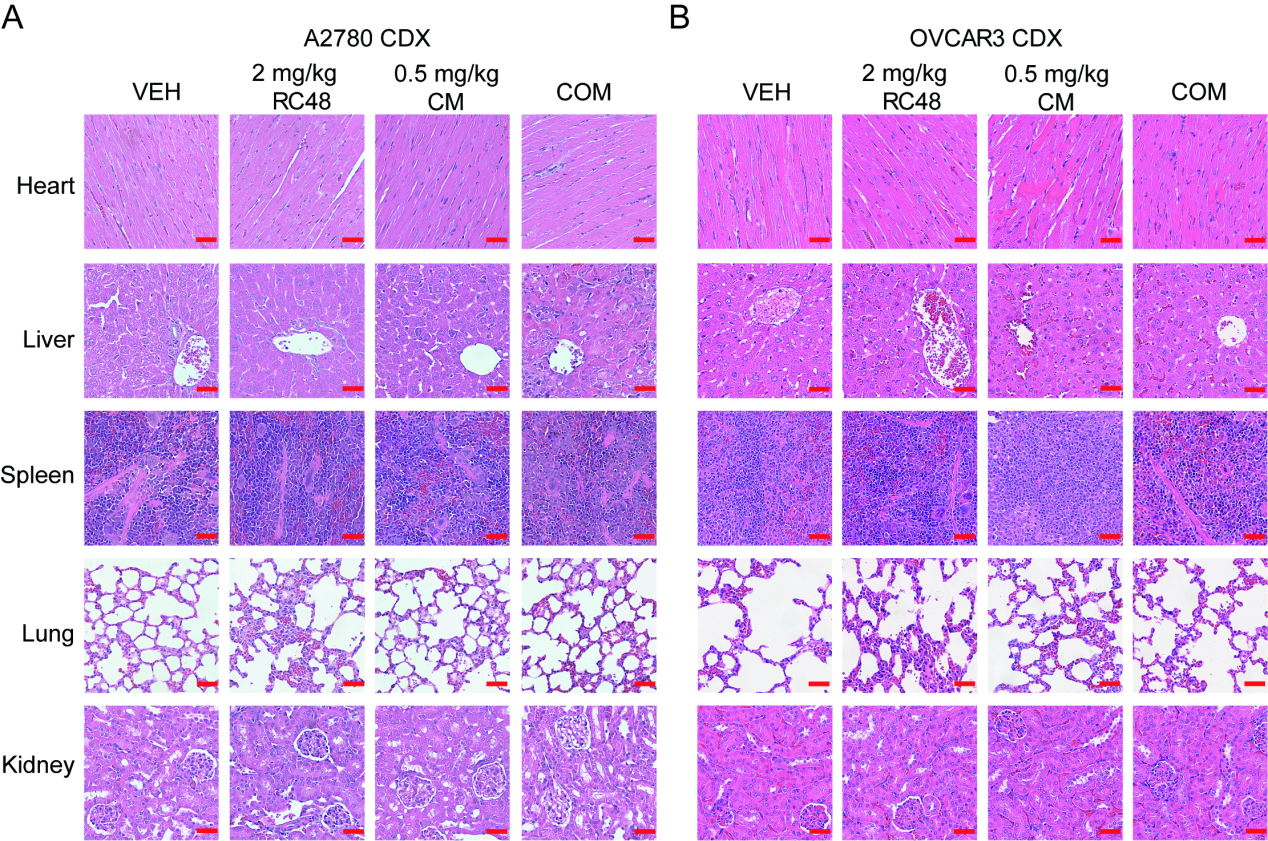


# Supplementary Figure 10. Combined effects of RC48 and CM on histology of the OC CDX models.

Treatment of RC48, CM, and their combination had no effects on heart, liver, spleen, lung and kidney in human A2780 (A) and OVCAR-3 (B) CDX models, proved by H&E staining assay for the evaluation of pathological changes in these organs of the CDX models. Images were captured at 400 × magnification. Scale bars = 20 µm.

**
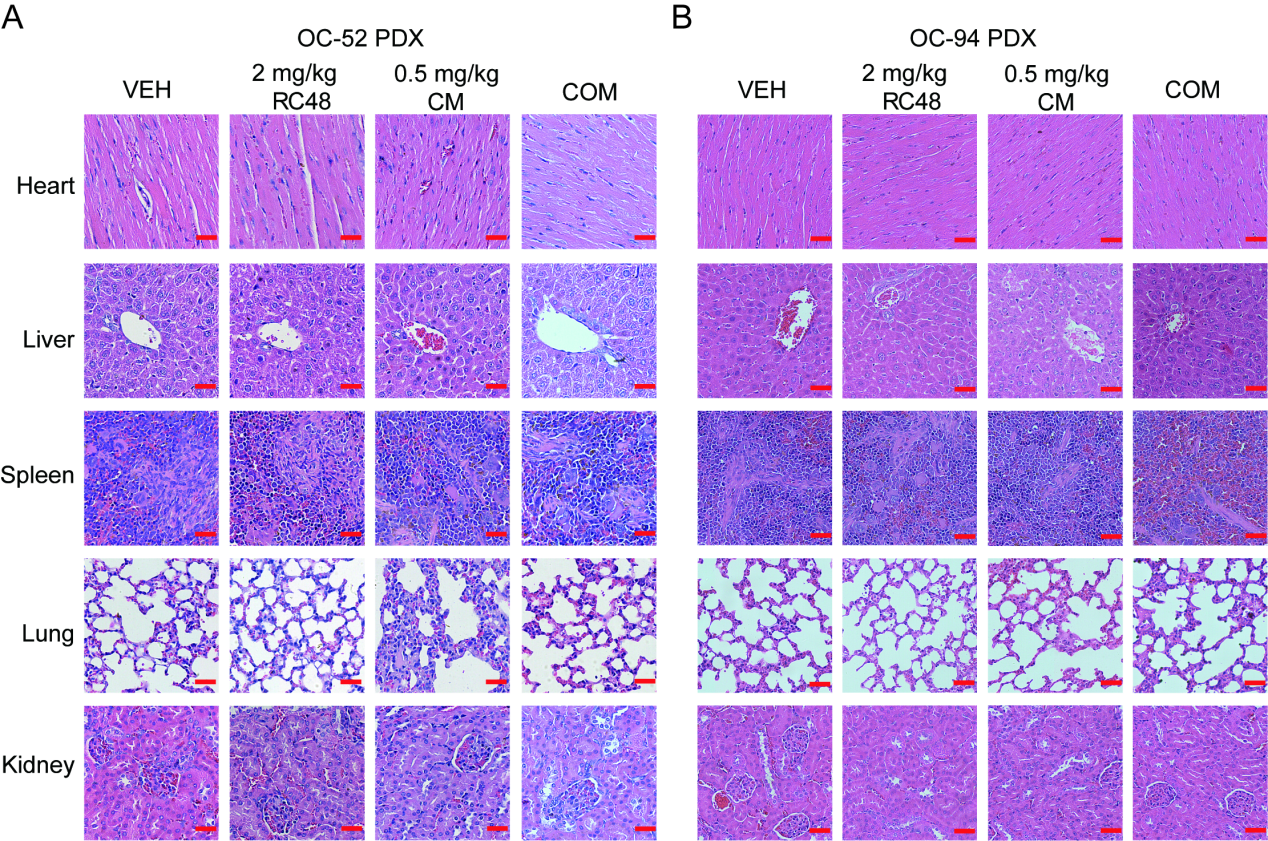
**

# Supplementary Figure 11. Combined effects of RC48 and CM on histology of the OC PDX models.

Treatment of RC48, CM, and their combination had no effects on heart, liver, spleen, lung and kidney in OC-52 (A) and OC-94 (B) PDX models, proved by H&E staining assay for the evaluation of pathological changes in these organs of the PDX models. Images were captured at 400 × magnification. Scale bars = 20 µm.

**Supplementary Tables**

# Supplemental Table 1. List of antibodies used for Western blot analysis

| Target | Company | Cat No. | Application | Dilution |
| --- | --- | --- | --- | --- |
| HER2 | CST | 2165 | WB | 1:2000 |
| P-HER2 | CST | 2243 | WB | 1:2000 |
| AKT | CST | 9272 | WB | 1:2000 |
| Phospho-Akt (Ser473) | CST | 4060 | WB | 1:2000 |
| mTOR | proteintech | 28273-1-AP | WB | 1:2000 |
| Phospho-mTOR (Ser2448) | CST | 2971 | WB | 1:1000 |
| MCL-1 | CST | 94296 | WB | 1:1000 |
| c-Myc | CST | 18583 | WB | 1:1000 |
| SNAI1 | Proteintech | 13099-1-AP | WB | 1:1000 |
| ZEB1 | Proteintech | 21544-1-AP | WB | 1:1000 |
| N-cadherin | Proteintech | 22018-1-AP | WB | 1:1000 |
| VEGFR2 | Proteintech | 26415-1-AP | WB | 1:1000 |
| VEGFR3 | Proteintech | 20712-1-AP | WB | 1:1000 |
| p-FOXO3A | Proteintech | 28755-1-AP | WB | 1:1000 |
| CDC25C | Proteintech | 16485-1-AP | WB | 1:1000 |
| Cyclin D3 | Proteintech | 26755-1-AP | WB | 1:1000 |
| GAPDH | Affinity | AF7021 | WB | 1:10000 |
| β-Tubulin | Proteintech | 10094-1-AP | WB | 1:20000 |

# Supplemental Table 2. List of antibodies used for immunofluorescence and immunohistochemistry analysis

| Target | Company | Cat No. | Application | Dilution |
| --- | --- | --- | --- | --- |
| Ki67 | CST | 9027 | IHC | 1:1000 |
| HER2 | Proteintech | 18299-1-AP | IHC/IF | 1:1000/1:200 |
| VEGFR2 | Proteintech | 26415-1-AP | IHC/IF | 1:1000/1:200 |
| VEGFR3 | Affinity | AF4201 | IF | 1:200 |
| E-cadherin | Proteintech | 20874-1-AP | IF | 1:500 |
| N-cadherin | Proteintech | 22018-1-AP | IF | 1:500 |

Supplemental Table 3. Patient clinical characteristics summary

| Primary tumor samples (n=90) | | | |
| --- | --- | --- | --- |
| Patient clinical characteristics | | | |
|  |  | All collected | |
| Parameters | Class | Number | Frequency (%) |
| Age |  |  |  |
|  | <30 y | 5 | 5.6 |
|  | ≥30 and <40 y | 5 | 5.6 |
|  | ≥40 and <50 y | 25 | 27.8 |
|  | ≥50 and <60 y | 39 | 43.3 |
|  | ≥60 and <70 y | 12 | 13.3 |
|  | >70 and <80 y | 4 | 4.4 |
| Subtype |  |  |  |
|  | High-grade serous adenocarcinoma | 68 | 75.6 |
|  | Low-grade serous adenocarcinoma | 10 | 11.1 |
|  | clear cell adenocarcinoma | 2 | 2.2 |
|  | mucinous cystadenocarcinoma | 5 | 5.6 |
|  | serous -mucinous adenocarcinoma | 1 | 1.1 |
|  | Endometrioid adenocarcinoma | 3 | 3.3 |
|  | carcinosarcoma | 1 | 1.1 |
| Stage |  |  |  |
|  | I | 19 | 21.1 |
|  | II | 9 | 10 |
|  | III | 50 | 55.6 |
|  | IV | 12 | 13.3 |

Supplemental Table 4 Patient clinical characteristics

| Number | Gender | Age | Immunohistochemical results | Clinical stage |
| --- | --- | --- | --- | --- |
| 1 | female | 42 | High-grade serous adenocarcinoma | IIIC1 |
| 2 | female | 47 | High-grade serous adenocarcinoma | IV |
| 3 | female | 45 | High-grade serous adenocarcinoma | IIIC1 |
| 4 | female | 58 | High-grade serous adenocarcinoma | IIIC |
| 5 | female | 53 | High-grade serous adenocarcinoma | IIIB |
| 6 | female | 58 | Clear cell carcinoma | IIIC |
| 7 | female | 54 | Mucinous cystadenocarcinoma | IA |
| 8 | female | 56 | High-grade serous adenocarcinoma | IIIC |
| 9 | female | 43 | High-grade serous adenocarcinoma | IIIC |
| 10 | female | 48 | High-grade serous adenocarcinoma | IC3 |
| 11 | female | 62 | High-grade serous adenocarcinoma | IIIC |
| 12 | female | 44 | High-grade serous adenocarcinoma | IIB |
| 13 | female | 53 | High-grade serous adenocarcinoma | IV |
| 14 | female | 52 | High-grade serous adenocarcinoma | IIIC |
| 15 | female | 51 | High-grade serous adenocarcinoma | IIIC |
| 16 | female | 32 | High-grade serous adenocarcinoma | IIIB |
| 17 | female | 51 | High-grade serous adenocarcinoma | IIIC |
| 18 | female | 38 | Low-grade serous adenocarcinoma | IIIA |
| 19 | female | 58 | High-grade serous adenocarcinoma | IIIC |
| 20 | female | 40 | High-grade serous adenocarcinoma | IA |
| 21 | female | 48 | High-grade serous adenocarcinoma | IC1 |
| 22 | female | 43 | High-grade serous adenocarcinoma | IIA |
| 23 | female | 43 | Clear cell carcinoma | IC1 |
| 24 | female | 50 | High-grade serous adenocarcinoma | IIB |
| 25 | female | 57 | High-grade serous adenocarcinoma | IVB |
| 26 | female | 53 | High-grade serous adenocarcinoma | IIIC |
| 27 | female | 66 | High-grade serous adenocarcinoma | IIB |
| 28 | female | 43 | Endometrioid adenocarcinoma | IC1 |
| 29 | female | 62 | High-grade serous adenocarcinoma | IIIC |
| 30 | female | 44 | serous -Mucinous adenocarcinoma | IC |
| 31 | female | 43 | High-grade serous adenocarcinoma | IV |
| 32 | female | 45 | High-grade serous adenocarcinoma | IIIC |
| 33 | female | 60 | Low-grade serous adenocarcinoma | IIB |
| 34 | female | 63 | High-grade serous adenocarcinoma | IV |
| 35 | female | 62 | High-grade serous adenocarcinoma | IIIC |
| 36 | female | 44 | High-grade serous adenocarcinoma | IIIC |
| 37 | female | 71 | High-grade serous adenocarcinoma | IVB |
| 38 | female | 42 | High-grade serous adenocarcinoma | II |
| 39 | female | 68 | High-grade serous adenocarcinoma | IIIC |
| 40 | female | 51 | High-grade serous adenocarcinoma | IIIC |
| 41 | female | 48 | High-grade serous adenocarcinoma | IIIC |
| 42 | female | 54 | High-grade serous adenocarcinoma | IIIB |
| 43 | female | 72 | High-grade serous adenocarcinoma | IIIC |
| 44 | female | 43 | High-grade serous adenocarcinoma | IIA |
| 45 | female | 54 | High-grade serous adenocarcinoma | IIIC |
| 46 | female | 69 | Low-grade serous adenocarcinoma | III |
| 47 | female | 52 | High-grade serous adenocarcinoma | IIIC |
| 48 | female | 58 | High-grade serous adenocarcinoma | IIIC |
| 49 | female | 54 | High-grade serous adenocarcinoma | IIIC |
| 50 | female | 40 | High-grade serous adenocarcinoma | IC |
| 51 | female | 51 | High-grade serous adenocarcinoma | IIIC |
| 52 | female | 50 | High-grade serous adenocarcinoma | IC |
| 53 | female | 74 | High-grade serous adenocarcinoma | IIIC |
| 54 | female | 52 | High-grade serous adenocarcinoma | IIIC |
| 55 | female | 55 | High-grade serous adenocarcinoma | IVB |
| 56 | female | 18 | Mucinous cystadenocarcinoma | IC |
| 57 | female | 55 | High-grade serous adenocarcinoma | IIIC |
| 58 | female | 22 | High-grade serous adenocarcinoma | IVB |
| 59 | female | 36 | High-grade serous adenocarcinoma | IC |
| 60 | female | 42 | High-grade serous adenocarcinoma | IIIC |
| 61 | female | 36 | Endometrioid adenocarcinoma | IIB |
| 62 | female | 54 | Low-grade serous adenocarcinoma | IVB |
| 63 | female | 40 | Low-grade serous adenocarcinoma | IIIC |
| 64 | female | 53 | High-grade serous adenocarcinoma | IVB |
| 65 | female | 54 | Low-grade serous adenocarcinoma | IIIC |
| 66 | female | 59 | low-grade serous adenocarcinoma was accompanied by focal high-grade serous adenocarcinoma | IIIB |
| 67 | female | 34 | Low-grade serous adenocarcinoma | IIIC |
| 68 | female | 12 | Mucinous cystadenocarcinoma | IC |
| 69 | female | 52 | High-grade serous adenocarcinoma | IIIC |
| 70 | female | 67 | High-grade serous adenocarcinoma | IIIC |
| 71 | female | 52 | High-grade serous adenocarcinoma | IC |
| 72 | female | 49 | Low-grade serous adenocarcinoma | IC2 |
| 73 | female | 51 | High-grade serous adenocarcinoma | IIIB |
| 74 | female | 20 | Mucinous cystadenocarcinoma | IC |
| 75 | female | 55 | High-grade serous adenocarcinoma | IIIC |
| 76 | female | 57 | High-grade serous adenocarcinoma | IVB |
| 77 | female | 67 | carcinosarcoma | IIIC |
| 78 | female | 47 | High-grade serous adenocarcinoma | IIIC |
| 79 | female | 55 | High-grade serous adenocarcinoma | IC |
| 80 | female | 51 | High-grade serous adenocarcinoma | IIIC |
| 81 | female | 56 | Low-grade serous adenocarcinoma | IIIC |
| 82 | female | 29 | Low-grade serous adenocarcinoma | IA |
| 83 | female | 48 | High-grade serous adenocarcinoma | IV |
| 84 | female | 67 | High-grade serous adenocarcinoma | IIB |
| 85 | female | 51 | High-grade serous adenocarcinoma | IIIC |
| 86 | female | 74 | High-grade serous adenocarcinoma | IIIB |
| 87 | female | 46 | High-grade serous adenocarcinoma | IIIC |
| 88 | female | 60 | Mucinous cystadenocarcinoma | IC |
| 89 | female | 56 | High-grade serous adenocarcinoma | IIIC |
| 90 | female | 50 | Endometrioid adenocarcinoma | IA |

#

# Supplemental Table 5. List of regulated KEGG pathways of combination treatment in A2780

| KEGGID | Description | GeneRatio | P value | Count | Up | Down |
| --- | --- | --- | --- | --- | --- | --- |
| hsa03030 | DNA replication | 30/2315 | 2.34E-09 | 30 | 1 | 29 |
| hsa04110 | Cell cycle | 74/2315 | 4.33E-09 | 74 | 12 | 62 |
| hsa04115 | p53 signaling pathway | 45/2315 | 1.71E-08 | 45 | 31 | 14 |
| hsa03420 | Nucleotide excision repair | 25/2315 | 0.000186351 | 25 | 8 | 17 |
| hsa04210 | Apoptosis | 62/2315 | 0.000319254 | 62 | 33 | 29 |
| hsa03410 | Base excision repair | 23/2315 | 0.001027414 | 23 | 2 | 21 |
| hsa04152 | AMPK signaling pathway | 44/2315 | 0.003396636 | 44 | 15 | 29 |
| hsa04012 | ErbB signaling pathway | 34/2315 | 0.004429574 | 34 | 16 | 18 |
| hsa04068 | FoxO signaling pathway | 50/2315 | 0.004741604 | 50 | 28 | 22 |
| hsa04150 | mTOR signaling pathway | 57/2315 | 0.012686363 | 57 | 32 | 25 |
| hsa04218 | Cellular senescence | 60/2315 | 0.022481034 | 60 | 26 | 34 |
| hsa03430 | \| Mismatch repair \| 14/2315 \|  \| \| --- \| --- \| --- \| | 14/2315 | 0.029584 | 14 | 0 | 14 |
| hsa04010 | MAPK signaling pathway | 92/2315 | 0.049392965 | 92 | 37 | 55 |
| hsa04510 | Focal adhesion | 73/2315 | 0.051054532 | 73 | 38 | 35 |
| hsa04151 | PI3K-Akt signaling pathway | 100/2315 | 0.051215453 | 100 | 43 | 57 |

#

# Supplemental Table 6. List of regulated KEGG pathways of combination treatment in OVCAR-3

| KEGGID | Description | GeneRatio | P value | Count | Up | Down |
| --- | --- | --- | --- | --- | --- | --- |
| hsa04512 | ECM-receptor interaction | 29/1128 | 1.60E-07 | 29 | 5 | 24 |
| hsa04350 | TGF-beta signaling pathway | 27/1128 | 2.28E-05 | 27 | 7 | 20 |
| hsa04068 | FoxO signaling pathway | 35/1128 | 0.000272889 | 35 | 13 | 22 |
| hsa04510 | Focal adhesion | 50/1128 | 0.00030806 | 50 | 11 | 39 |
| hsa04668 | TNF signaling pathway | 30/1128 | 0.000345353 | 30 | 6 | 24 |
| hsa04151 | PI3K-Akt signaling pathway | 68/1128 | 0.000373061 | 68 | 17 | 51 |
| hsa04115 | p53 signaling pathway | 20/1128 | 0.00228657 | 20 | 8 | 12 |
| hsa04010 | MAPK signaling pathway | 56/1128 | 0.003488012 | 56 | 18 | 38 |
| hsa04514 | Cell adhesion molecules | 28/1128 | 0.006585891 | 28 | 8 | 20 |
| hsa04012 | ErbB signaling pathway | 20/1128 | 0.00745659 | 20 | 8 | 12 |
| hsa04630 | JAK-STAT signaling pathway | 26/1128 | 0.023358844 | 26 | 4 | 22 |
| hsa04064 | NF-κB signaling pathway | 19/1128 | 0.03060001 | 19 | 0 | 19 |
| hsa04540 | Gap junction | 19/1128 | 0.03060001 | 19 | 7 | 12 |
| hsa04520 | Adherens junction | 19/1128 | 0.033465293 | 19 | 6 | 13 |
| hsa04110 | Cell cycle | 28/1128 | 0.04358134 | 28 | 16 | 12 |

#

# Supplemental Table 7. Regulated genes correlated with apoptosis

| Cell line | Gene symbol | log2FoldChange | P value | padj |
| --- | --- | --- | --- | --- |
| A2780 | TUBA1B | -1.268824571 | 3.00651E-22 | 3.91856E-20 |
|  | BIRC5 | -0.938697752 | 1.73909E-16 | 1.49622E-14 |
|  | PARP1 | -0.657529208 | 4.62248E-11 | 2.17605E-09 |
|  | LMNB2 | -0.974576766 | 5.39793E-10 | 2.20784E-08 |
|  | ITPR3 | -0.644507406 | 1.02336E-05 | 0.000164879 |
|  | TNFRSF10B | 0.919635174 | 2.43798E-19 | 2.66121E-17 |
|  | FAS | 1.609451434 | 6.11934E-19 | 6.47723E-17 |
|  | PIK3R3 | 0.611188289 | 1.02707E-07 | 2.7261E-06 |
|  | CASP10 | 1.380339768 | 4.80106E-07 | 1.1004E-05 |
|  | CAPN2 | 0.500304025 | 1.20015E-06 | 2.47178E-05 |
| OVCAR-3 | TNFSF10 | -1.090650026 | 0.00137541 | 0.03997253 |
|  | XIAP | -0.674825378 | 0.029059769 | 0.252105293 |
|  | ACTBP7 | -1.353198354 | 0.049707872 | 0.317533433 |
|  | TUBA1A | 0.930165604 | 1.08456E-07 | 1.79269E-05 |
|  | CASP9 | 0.631918742 | 0.003549028 | 0.077463523 |
|  | CASP6 | 0.546836777 | 0.004745501 | 0.092730503 |
|  | CASP7 | 0.473079254 | 0.005445918 | 0.101071148 |
|  | ENDOG | 0.894514613 | 0.00668857 | 0.115614623 |
|  | CTSF | 0.912776323 | 0.021482318 | 0.219383111 |
|  | MAPK3 | 0.615506637 | 0.034305946 | 0.272497095 |

# Supplemental Table 8. Regulated genes correlated with cell cycle

| Cell line | Gene symbol | log2FoldChange | P value | padj |
| --- | --- | --- | --- | --- |
| A2780 | E2F2 | -1.736181108 | 1.23376E-32 | 3.42025E-30 |
|  | CDC45 | -1.396906013 | 7.64036E-24 | 1.21308E-21 |
|  | E2F1 | -1.703604061 | 8.37434E-24 | 1.31764E-21 |
|  | MCM7 | -1.2615604 | 1.47335E-22 | 2.02615E-20 |
|  | MCM5 | -1.46684745 | 1.51639E-20 | 1.75389E-18 |
|  | ESPL1 | -1.157333604 | 1.04336E-19 | 1.17563E-17 |
|  | MCM2 | -1.320438136 | 9.81004E-18 | 9.26121E-16 |
|  | PKMYT1 | -1.264000964 | 4.91078E-12 | 2.67373E-10 |
|  | MDM2 | 2.198986486 | 9.23279E-57 | 6.45002E-54 |
|  | SMAD3 | 1.475177907 | 2.55998E-22 | 3.38713E-20 |
| OVCAR-3 | MYC | -1.115377522 | 4.0463E-05 | 0.00266418 |
|  | CDK6 | -1.583711169 | 7.71732E-05 | 0.004404979 |
|  | TGFB2 | -0.685126439 | 0.005296328 | 0.09945814 |
|  | MCM6 | -0.375050576 | 0.007265487 | 0.121998673 |
|  | PRKDC | -1.003146717 | 0.017141918 | 0.193498555 |
|  | GSK3B | -0.540327815 | 0.035149545 | 0.274275597 |
|  | CCNA2 | 0.678485543 | 8.63271E-05 | 0.004806452 |
|  | ESPL1 | 0.526678854 | 0.000244648 | 0.01115538 |
|  | PTTG1 | 0.920884289 | 0.001044069 | 0.032144993 |
|  | CDKN1B | 0.690678131 | 0.003995626 | 0.083644582 |

# Supplemental Table 9. Regulated genes correlated with cell senescence

| Cell line | Gene symbol | log2FoldChange | P value | padj |
| --- | --- | --- | --- | --- |
| A2780 | E2F2 | -1.736181108 | 1.23376E-32 | 3.42025E-30 |
|  | MYBL2 | -1.523769686 | 4.89252E-30 | 1.18678E-27 |
|  | E2F1 | -1.703604061 | 8.37434E-24 | 1.31764E-21 |
|  | FOXM1 | -0.863903503 | 6.83154E-12 | 3.61554E-10 |
|  | EIF4EBP1 | -1.040960425 | 2.88287E-09 | 1.04459E-07 |
|  | MDM2 | 2.198986486 | 9.23279E-57 | 6.45002E-54 |
|  | SMAD3 | 1.475177907 | 2.55998E-22 | 3.38713E-20 |
|  | PIK3R3 | 0.611188289 | 1.02707E-07 | 2.7261E-06 |
|  | TRPM7 | 0.698401954 | 7.33832E-07 | 1.59606E-05 |
|  | CAPN2 | 0.500304025 | 1.20015E-06 | 2.47178E-05 |
| OVCAR-3 | TGFBR2 | -0.867756073 | 3.00824E-09 | 7.14465E-07 |
|  | MYC | -1.115377522 | 4.0463E-05 | 0.00266418 |
|  | CDK6 | -1.583711169 | 7.71732E-05 | 0.004404979 |
|  | ZFP36L1 | -0.670724553 | 0.000108894 | 0.005798441 |
|  | TGFB2 | -0.685126439 | 0.005296328 | 0.09945814 |
|  | PPP3CA | -1.007919339 | 0.012405947 | 0.16367671 |
|  | FOXO3 | -0.643631099 | 0.014105217 | 0.175271407 |
|  | CCNA2 | 0.678485543 | 8.63271E-05 | 0.004806452 |
|  | SLC25A5-AS1 | 1.629812994 | 0.000700057 | 0.024201532 |
|  | MAPK3 | 0.615506637 | 0.034305946 | 0.272497095 |

# Supplemental Table 10. Regulated genes correlated with focal adhesion

| Cell line | Gene symbol | log2FoldChange | P value | padj |
| --- | --- | --- | --- | --- |
| A2780 | PDGFRB | -1.035717849 | 2.7067E-09 | 9.91037E-08 |
|  | FLT4 | -1.106990598 | 7.23416E-08 | 1.96799E-06 |
|  | VEGFB | -0.939904954 | 1.92933E-07 | 4.85529E-06 |
|  | COL9A1 | -1.259628037 | 1.12052E-05 | 0.000178557 |
|  | COL4A5 | 0.91831275 | 5.22557E-14 | 3.52373E-12 |
|  | VCL | 0.677330256 | 4.10539E-12 | 2.26901E-10 |
|  | ITGAV | 0.70714208 | 4.41997E-08 | 1.25316E-06 |
|  | PIK3R3 | 0.611188289 | 1.02707E-07 | 2.7261E-06 |
|  | GSK3B | 0.660803959 | 6.38853E-07 | 1.4157E-05 |
|  | CAPN2 | 0.500304025 | 1.20015E-06 | 2.47178E-05 |
| OVCAR-3 | ITGA9 | -1.479886822 | 1.43754E-06 | 0.00017281 |
|  | ITGAV | -1.019601984 | 4.32805E-05 | 0.00279804 |
|  | ITGA2 | -1.225808359 | 0.000355971 | 0.014748187 |
|  | EGFR | -1.016219855 | 0.000957731 | 0.030721193 |
|  | RELN | -2.379430804 | 0.002313084 | 0.057082455 |
|  | LAMA4 | -1.206381902 | 0.002699229 | 0.064311365 |
|  | DOCK1 | -0.57077159 | 0.004996615 | 0.0958469 |
|  | LAMC1 | -0.56152524 | 0.00730032 | 0.122195654 |
|  | ITGB4 | -0.386262552 | 0.015448496 | 0.185288542 |
|  | LAMB1 | -0.480080011 | 0.027679358 | 0.246953038 |

# Supplemental Table 11. Regulated genes in PI3K-AKT signaling pathway

| Cell line | Gene symbol | log2FoldChange | P value | padj |
| --- | --- | --- | --- | --- |
| A2780 | PKN3 | -1.486342212 | 1.37055E-17 | 1.27323E-15 |
|  | FGFR4 | -1.51953731 | 5.22808E-15 | 3.80452E-13 |
|  | ERBB3 | -1.415374592 | 1.00297E-09 | 3.91002E-08 |
|  | PDGFRB | -1.035717849 | 2.7067E-09 | 9.91037E-08 |
|  | EIF4EBP1 | -1.040960425 | 2.88287E-09 | 1.04459E-07 |
|  | EFNA3 | -2.446230089 | 2.42988E-08 | 7.22963E-07 |
|  | FLT4 | -1.106990598 | 7.23416E-08 | 1.96799E-06 |
|  | MDM2 | 2.198986486 | 9.23279E-57 | 6.45002E-54 |
|  | COL4A5 | 0.91831275 | 5.22557E-14 | 3.52373E-12 |
|  | ITGAV | 0.70714208 | 4.41997E-08 | 1.25316E-06 |
| OVCAR-3 | ITGA9 | -1.479886822 | 1.43754E-06 | 0.00017281 |
|  | MYC | -1.115377522 | 4.0463E-05 | 0.00266418 |
|  | ITGAV | -1.019601984 | 4.32805E-05 | 0.00279804 |
|  | CDK6 | -1.583711169 | 7.71732E-05 | 0.004404979 |
|  | ITGA2 | -1.225808359 | 0.000355971 | 0.014748187 |
|  | EGFR | -1.016219855 | 0.000957731 | 0.030721193 |
|  | KIT | -1.427468187 | 0.001874902 | 0.049175103 |
|  | RELN | -2.379430804 | 0.002313084 | 0.057082455 |
|  | LAMA4 | -1.206381902 | 0.002699229 | 0.064311365 |
|  | CDKN1B | 0.690678131 | 0.003995626 | 0.083644582 |

# Supplemental Table 12. Regulated genes in FOXO signaling pathway

| Cell line | Gene symbol | log2FoldChange | P value | padj |
| --- | --- | --- | --- | --- |
| A2780 | PLK1 | -0.889786793 | 2.36222E-11 | 1.14283E-09 |
|  | PCK2 | -0.563644839 | 4.93777E-06 | 8.67077E-05 |
|  | AKT1 | -0.652419085 | 2.50752E-05 | 0.000365252 |
|  | MAPK3 | -0.647592378 | 2.94805E-05 | 0.000420558 |
|  | MDM2 | 2.198986486 | 9.23279E-57 | 6.45002E-54 |
|  | PRKAB2 | 1.4784797 | 2.59286E-26 | 5.08812E-24 |
|  | STAT3 | 1.134092161 | 2.29419E-22 | 3.05863E-20 |
|  | SMAD3 | 1.475177907 | 2.55998E-22 | 3.38713E-20 |
|  | PRKAB1 | 1.004958958 | 3.09538E-14 | 2.12838E-12 |
|  | PIK3R3 | 0.611188289 | 1.02707E-07 | 2.7261E-06 |
| OVCAR-3 | TGFBR2 | -0.867756073 | 3.00824E-09 | 7.14465E-07 |
|  | EGFR | -1.016219855 | 0.000957731 | 0.030721193 |
|  | TNFSF10 | -1.090650026 | 0.00137541 | 0.03997253 |
|  | STK4 | -0.526375414 | 0.002545959 | 0.061490529 |
|  | TGFB2 | -0.685126439 | 0.005296328 | 0.09945814 |
|  | FOXO3 | -0.643631099 | 0.014105217 | 0.175271407 |
|  | BNIP3P11 | -1.150072029 | 0.041274475 | 0.29406147 |
|  | CDKN1B | 0.690678131 | 0.003995626 | 0.083644582 |
|  | ARAF | 0.545560606 | 0.011369836 | 0.15553151 |
|  | MAPK3 | 0.615506637 | 0.034305946 | 0.272497095 |

# Supplemental Table 13. Regulated genes in MAPK signaling pathway

| Cell line | Gene symbol | log2FoldChange | P value | padj |
| --- | --- | --- | --- | --- |
| A2780 | FGFR4 | -1.51953731 | 5.22808E-15 | 3.80452E-13 |
|  | RASGRP2 | -1.571446524 | 8.8007E-12 | 4.57453E-10 |
|  | ERBB3 | -1.415374592 | 1.00297E-09 | 3.91002E-08 |
|  | PDGFRB | -1.035717849 | 2.7067E-09 | 9.91037E-08 |
|  | EFNA3 | -2.446230089 | 2.42988E-08 | 7.22963E-07 |
|  | FLT4 | -1.106990598 | 7.23416E-08 | 1.96799E-06 |
|  | ARRB2 | -0.751493324 | 1.45424E-07 | 3.75714E-06 |
|  | VEGFB | -0.939904954 | 1.92933E-07 | 4.85529E-06 |
|  | FAS | 1.609451434 | 6.11934E-19 | 6.47723E-17 |
|  | PLA2G4C | 1.267127765 | 1.51911E-10 | 6.64944E-09 |
| OVCAR-3 | TGFBR2 | -0.867756073 | 3.00824E-09 | 7.14465E-07 |
|  | MYC | -1.115377522 | 4.0463E-05 | 0.00266418 |
|  | EGFR | -1.016219855 | 0.000957731 | 0.030721193 |
|  | KIT | -1.427468187 | 0.001874902 | 0.049175103 |
|  | STK4 | -0.526375414 | 0.002545959 | 0.061490529 |
|  | RASA1 | -0.650789644 | 0.004540687 | 0.090290253 |
|  | TGFB2 | -0.685126439 | 0.005296328 | 0.09945814 |
|  | EFNA5 | -1.13068043 | 0.006575902 | 0.114414922 |
|  | CACNG4 | 0.420829666 | 0.003000071 | 0.068992944 |
|  | MEF2C | 0.859130354 | 0.010537085 | 0.149826211 |

# Supplemental Table 14. Regulated genes in p53 signaling pathway

| Cell line | Gene symbol | log2FoldChange | P value | padj |
| --- | --- | --- | --- | --- |
| A2780 | GTSE1 | -0.851916547 | 7.70899E-13 | 4.65874E-11 |
|  | TP53I3 | 2.627571736 | 2.4289E-77 | 3.85643E-74 |
|  | ZMAT3 | 2.23278956 | 1.87045E-58 | 1.42032E-55 |
|  | MDM2 | 2.198986486 | 9.23279E-57 | 6.45002E-54 |
|  | SESN1 | 1.889017945 | 1.90846E-46 | 1.0416E-43 |
|  | RRM2B | 1.830295149 | 1.06758E-42 | 4.78083E-40 |
|  | TNFRSF10B | 0.919635174 | 2.43798E-19 | 2.66121E-17 |
|  | PPM1D | 1.092004215 | 4.22383E-19 | 4.54819E-17 |
|  | FAS | 1.609451434 | 6.11934E-19 | 6.47723E-17 |
|  | EI24 | 0.931700673 | 4.36714E-16 | 3.59774E-14 |
| OVCAR-3 | THBS1 | -1.951252845 | 2.94742E-09 | 7.14465E-07 |
|  | CDK6 | -1.583711169 | 7.71732E-05 | 0.004404979 |
|  | MDM4 | -0.825310218 | 0.00583261 | 0.106626567 |
|  | ATR | -0.702262446 | 0.048946721 | 0.31546977 |
|  | CCNB1 | 0.912642281 | 1.60502E-09 | 4.22517E-07 |
|  | CCNB2 | 0.75823731 | 1.52134E-05 | 0.001228142 |
|  | CDKN1A | 1.284068521 | 5.97109E-05 | 0.003602631 |
|  | CASP9 | 0.631918742 | 0.003549028 | 0.077463523 |
|  | PERP | 0.609128479 | 0.005254077 | 0.099133998 |
|  | AIFM2 | 0.667258357 | 0.014272166 | 0.176792137 |

# Supplemental Table 15. Gene set enrichment analysis (GSEA) analysis

| GSEA pathway | NES | NES | FDR q VALUE | FDR q VALUE |
| --- | --- | --- | --- | --- |
|  | A2780 | OVCAR-3 | A2780 | OVCAR-3 |
| PLATINUM DRUG RESISTANCE | 1.79 | 1.53 | 0.048 | 0.179 |
| LYSOSOME | 1.52 | 1.59 | 0.214 | 0.202 |
| FERROPTOSIS | 1.45 | 1.55 | 0.243 | 0.175 |
| LONG_TERM DEPRESSION | 1.39 | 1.64 | 0.276 | 0.206 |
| AUTOPHAGY _ OTHER | 1.36 | 1.55 | 0.296 | 0.171 |
| TGF_BETA SIGNALING PATHWAY | -1.41 | -1.67 | 0.236 | 0.102 |
| GAP JUNCTION | -1.42 | -1.57 | 0.244 | 0.134 |
| HIPPO SIGNALING PATHWAY | -1.48 | -1.6 | 0.216 | 0.128 |
| CELLULAR SENESCENCE | -1.5 | -1.55 | 0.210 | 0.149 |

# 
